# Tomato NAC2-DREB2 module fine-tunes saline–alkali stress sensitivity via modulation of melatonin biosynthesis and ROS homeostasis

**DOI:** 10.1093/hr/uhag029

**Published:** 2026-01-30

**Authors:** Songchong Lu, Yan Sun, Xinshuang Zhang, Wenying Zhu, Xin Liu, Fu Wang, Sheng Luan, Aoxue Wang, Hui Wang

**Affiliations:** Shandong Key Laboratory for Germplasm Innovation of Saline–Alkaline Tolerant Grasses and Trees, College of Grassland Science, Qingdao Agricultural University, Qingdao 266109, China; Shandong Key Laboratory for Germplasm Innovation of Saline–Alkaline Tolerant Grasses and Trees, College of Grassland Science, Qingdao Agricultural University, Qingdao 266109, China; College of Life Sciences, Qingdao Agricultural University, Qingdao 266109, China; College of Horticulture, Qingdao Agricultural University, Qingdao 266109, China; College of Life Sciences, Qingdao Agricultural University, Qingdao 266109, China; College of Horticulture, Qingdao Agricultural University, Qingdao 266109, China; Department of Plant and Microbial Biology, University of California, Berkeley, CA 94720, USA; College of Horticulture and Landscape Architecture, Northeast Agricultural University, Harbin 150030, China; College of Horticulture, Qingdao Agricultural University, Qingdao 266109, China

## Abstract

Soil salinization poses a serious threat to plant development and represents a major obstacle to the sustainable production of crops worldwide. Melatonin (MT) contributes prominently to plant tolerance against abiotic environments. However, the molecular basis of transcriptional regulation underlying melatonin accumulation in tomato under saline–alkali stress is still largely unknown. Herein, we identify SlNAC2, a NAC transcription factor in tomato induced by saline–alkali stress, which suppresses the key melatonin biosynthetic genes *SlCOMT2* and *SlSNAT*, while activating *SlCV*, a gene linked to reactive oxygen species (ROS) accumulation and programmed cell death. These regulatory effects reduce MT levels and promote excessive ROS production, ultimately altering the plant’s tolerance to saline–alkali stress. Silencing of *SlNAC2* through the RNA interference method significantly improves saline–alkali tolerance in tomato, while its constitutive overexpression shows increased susceptibility to saline–alkali stress. Further evidence reveals that under saline–alkali conditions, SlNAC2 directly targets cis-elements of *SlCOMT2* and *SlSNAT* promoters, suppressing their transcription and consequently reducing melatonin levels, whereas simultaneously binding to the *SlCV* promoter to activate its expression, ultimately leading to ROS accumulation. Moreover, comprehensive protein interaction analyses confirmed that SlNAC2 physically associates with SlDREB2, a DREB-type transcription factor involved in salt stress response. Through its interaction with SlNAC2, SlDREB2 partially attenuates its repression of *SlCOMT2* and *SlSNAT*, thereby increasing melatonin accumulation and ROS scavenging, ultimately enhancing tomato’s resilience to saline–alkali stress conditions. Collectively, our findings reveal a SlNAC2–SlDREB2 regulatory module that finely tunes melatonin synthesis and ROS levels to regulate tomato’s response to saline–alkali stress, providing new strategies for developing stress-resilient tomato varieties.

## Introduction

Saline–alkali stress constitutes a widespread abiotic challenge that severely impairs plant growth and development, and even reduces crop productivity [1–3]. The Food and Agriculture Organization has reported that salinity and/or alkalinity affects over 1.4 billion hectares of global land, reflecting a critical abiotic barrier to sustainable agriculture. Typically, salinized soils are characterized by the accumulation of both neutral salts (NaCl, Na_2_SO_4_) and/or alkaline salts (NaHCO_3_, Na_2_CO_3_). Plants subjected to alkaline salts experience more pronounced damage than those exposed to neutral salts, primarily due to the disruption of cellular homeostasis caused by osmotic imbalance, elevated pH stress, toxic ion accumulation, and nutrient deprivation [4]. To date, extensive research has focused on plant adaptation to neutral salt stress; however, simultaneous exposure to both saline and alkaline conditions elicits synergistic stress responses that lead to more severe physiological damage [5, 6]. Therefore, elucidating the molecular mechanisms underlying plant tolerance to saline–alkali stress and developing resilient crop varieties hold significant potential for improving crop yields in salt- and alkali-affected regions. To adapt to saline–alkali conditions, plants have evolved sophisticated molecular networks that maintain physiological homeostasis and ensure proper developmental progression [7]. For instance, H^+^ efflux from roots acidifies the rhizosphere, thereby facilitating plant tolerance to high-pH stress [8]. The calcium-dependent SOS signaling cascade, including SOS1, SOS2, and SOS3, together with other ion-regulatory elements such as HKTs, NHXs, and CBL10, plays a critical role in maintaining intracellular ion homeostasis in plants [9–14]. These adaptive strategies contribute to promoting plant resilience and maintaining growth in salt–alkali environments.

In response to both intrinsic signals and environmental challenges, plants primarily rely on transcription regulation to fine-tune gene expression [15]. Multiple transcription factor families, such as NAC, WRKY, MYB, AP2/ERF, and bHLH, are involved in modulating plant responses to abiotic stress [16, 17]. NAC transcription factors—named after NAM, ATAF1/2, and CUC proteins—represent a large group of plant-specific transcription factors characterized by a conserved N-terminus and a highly variable C-terminus. The functional versatility of NAC transcription factors stems primarily from the structural diversity of their C-terminal domain, which imparts flexibility and specificity in regulating multiple biological pathways [18–20]. NAC transcription factors contribute significantly to orchestrating plant growth and stress adaptation, including flowering, lateral development, senescence, immune defense, and abiotic stress [18, 20–26]. In tomato, SlNAC3 functions as a transcriptional modulator that promotes ethylene burst under cold stress by activating the expression of genes responsible for its biosynthesis [27]. Additionally, LbNAM2, a NAC transcription factor in wolfberry, positively regulates drought resistance by activating the transcription of *LbZDS*, a crucial gene involved in carotenoid synthesis [28]. Alshareef *et al.* [29] reported that the NAC transcription factor AtJUB1 confers salt stress resistance by promoting the accumulation of osmoprotective compounds. In rice, ONAC022 facilitates salt stress adaptation primarily by upregulating the expression of *OsNCEDs* and *OsPSY*, two key genes in the ABA synthesis pathway, which boosts ABA production and strengthens the plant’s resistance to saline stress [30]. Moreover, OsNAC2 functions as a key transcription factor that contributes to the modulation of developmental processes and environmental stress responses. Although the function of OsNAC2 has been well characterized in rice [21, 22, 31–33], its contribution to stress tolerance in other crops like tomato is poorly defined, especially regarding its regulation of saline–alkali stress responses. Intriguingly, recent findings in alfalfa reveal that the MsNAC2a impairs salinity tolerance by suppressing hydrogen sulfide (H₂S) accumulation and enhancing reactive oxygen species (ROS) levels under salt stress [34]. Despite increasing interest in stress responses, the precise mechanisms through which NAC2 transcription factors modulate saline–alkali tolerance in tomato are still largely unknown and warrant further investigation.

Dehydration-Responsive Element-Binding (DREB) proteins represent an important subfamily of the AP2/ERF transcription factor family, which is categorized into the AP2, ERF, DREB, and RAV subfamilies based on the number and type of AP2 DNA-binding domains [35–38]. The DREB subfamily is characterized by a specific ability to bind the cis-acting dehydration-responsive element/C-repeat (DRE/CRT) motif and plays pivotal roles in the transcriptional regulation of genes involved in plant responses to dehydration, drought, salinity, and low temperature [39–44]. A growing body of evidence demonstrates that DREB is transcriptionally activated under a wide range of abiotic stresses, including cold, drought, oxidative stress, and salinity [45–48]. In particular, DREB2A has been shown to be involved in salt stress signaling and contributes significantly to salt tolerance in diverse plant species such as *Arabidopsis*, soybean, tomato, pumpkin, and rice [41, 43, 49, 50]. For example, in both *Arabidopsis* and tomato, SlDREB2, identified in tomato as a DREB-type transcription factor, confers increased salinity resistance through modulation of ABA signaling networks and maintenance of ion homeostasis (K^+^/Na^+^) [50]. CmoDREB2A from pumpkin improves the salt resistance in grafted cucumber by forming a regulatory complex with CmoNAC1, thereby fine-tuning ABA and ROS (H_2_O_2_) pathways and maintaining ion homeostasis [41]. Despite extensive studies on DREB modulators under salinity, the functional mechanisms of these factors in tomato, particularly under saline–alkali stress, are still poorly understood. In addition, accumulating evidence indicates that NAC and DREB transcription factors display overlapping functions in salt stress responses, suggesting that their potential interaction in tomato under saline–alkali stress warrants further investigation.

Melatonin (*N*-acetyl-5-methoxytryptamine), a product of tryptophan metabolism, is found in organisms ranging from animals to bacteria and plants [51]. In plants, melatonin, functioning as a multifunctional molecule, orchestrates a myriad of physiological processes, including root formation, seed development, fruit maturation, senescence, and biotic and abiotic stress [51–56]. In recent decades, a well-defined enzymatic pathway for melatonin production has been established in plants, with five major enzymes, such as tryptamine 5-hydroxylase (T5H), tryptophan decarboxylase (TDC), serotonin *N*-acetyltransferase (SNAT), caffeic acid *O*-methyltransferase (COMT), and *N*-acetylserotonin *O*-methyltransferase (ASMT), recognized as central to this pathway [57]. COMT, which is evolutionarily derived from ASMT, has been shown to have dramatically improved catalytic efficiency, often ranging from 10 to 100 times higher [58, 59]. Previous research has focused on and elucidated the functional significance of *COMT* genes in various biological processes. For instance, under saline–alkali stress, elevated nitric oxide (NO) levels upregulate the expression of *COMT*, resulting in melatonin accumulation that contributes to NO scavenging and alleviation of nitrosative stress. This melatonin, in turn, prevents S-nitrosylation of H^+^-ATPase 2 (HA2), thereby maintaining its activity and supporting stress adaptation [60]. Additionally, overexpression of *ClCOMT1* or melatonin application has been shown to improve low temperature tolerance in watermelon by triggering CBF-mediated cold-responsive mechanisms [61]. In citrus, functional studies of *PtCOMT5* indicated that its overexpression confers drought resistance by stimulating melatonin biosynthesis and root growth; in contrast, the *ptcomt5* mutant lines generated by CRISPR-Cas9 exhibit heightened drought sensitivity [62]. In tomato, MT delays leaf senescence primarily through suppressing the senescence-associated gene *SlCV*, thereby maintaining ROS homeostasis and stabilizing chloroplasts, while MT degradation accelerates aging, highlighting the central role of SlCV in MT-regulated ROS balance and senescence control [56]. A recent study revealed that during *Botrytis cinerea* infection, nitric oxide (NO) stabilizes the SlCOMT2 protein through S-nitrosylation, while jasmonic acid (JA)-activated transcription factor SlMYC2 directly targets *SlCOMT1* and *SlCOMT2*, thereby upregulating their expression. Collectively, in response to *B. cinerea* infection, NO and JA collaboratively enhance COMT-mediated melatonin accumulation by regulating COMT function at both the protein and gene expression levels [63]. In alfalfa, MsSNAT1, as a downstream target of MsbZIP55, is essential for melatonin biosynthesis and salinity stress resistance [51]. Although considerable progress has been made in elucidating *COMT* and *SNAT* genes’ roles in melatonin biosynthesis and stress tolerance, the upstream transcriptional mechanisms under saline–alkali stress in tomato remain largely unknown.

Tomato, a globally cultivated and economically important vegetable crop, remains highly sensitive to saline–alkali and other abiotic stress that continues to threaten sustainable production. Thus, unraveling the tolerance mechanisms of tomato under saline–alkali stress is essential for targeted breeding interventions focused on enhancing crop resistance [17, 60]. Herein, our findings indicate that SlNAC2 is a crucial component of the tomato’s defense mechanism against saline–alkali stress. The transcriptional level of *SlNAC2* was markedly upregulated in response to saline–alkali treatment; however, functional analysis revealed that silencing SlNAC2 conferred increased tolerance to saline–alkali stress in tomato, while overexpressing the gene compromised the plant’s stress resilience. Experimental evidence from molecular and biochemical studies demonstrated that SlNAC2 directly interacts with the promoter sequences of *SlCOMT2* and *SlSNAT*, thereby repressing melatonin biosynthesis, while simultaneously activating the ROS-associated gene *SlCV*, together resulting in reduced melatonin accumulation, elevated ROS levels, and heightened susceptibility to saline–alkali stress in tomato. In addition, both biochemical and genetic analyses confirmed that SlDREB2, a member of the DREB transcription factor family, physically interacts with SlNAC2, thereby modulating its downstream regulatory targets and fine-tuning tomato responses to saline–alkali stress. The established SlNAC2–SlDREB2–melatonin module uncovers a previously uncharacterized regulatory mechanism in the orchestration of salt–alkali stress responses, highlighting NAC transcription factors as critical modulators of stress resilience and potential candidates for crop improvement strategies.

## Results

### Isolation and identification of *SlNAC2*

**Figure 1:**
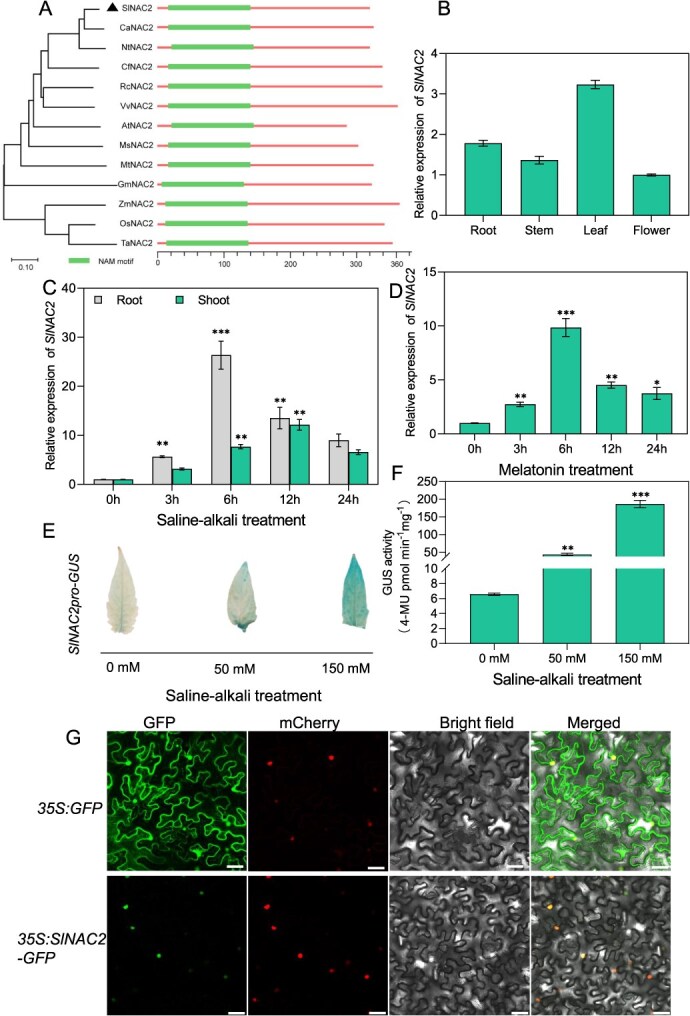
Characterization of SlNAC2 as a potential NAC transcription factor involved in saline–alkali tolerance. (A) Phylogenetic analysis of SlNAC2 (indicated by black triangle) with its homologs from different plant species, including *A. thaliana*, *Vigna radiata*, *Glycine max*, *C. annuum*, *N. tabacum*, *Rosa chinensis*, *Cornus florida*, *Medicago truncatula*, *Medicago sativa, Triticum aestivum*, *Oryza sativa*, and *Zea mays*. Phylogenetic tree of SlNAC2 and its homologous proteins was generated through the software DNAMAN v.9.0. (B) Tissue-specific expression analysis of *SlNAC2* in tomato, examining its levels in roots, stems, leaves, and flowers. Data were normalized against the flower. (C) Expression patterns of *SlNAC2* in leaves and roots under Na_2_CO_3_ treatment over the indicated time course. (D) Expression patterns of *SlNAC2* under Na_2_CO_3_ treatment over the indicated time course. *SlActin* was used as an internal control. Data shown are means ± SD of three biological replicates. Relative expression was calculated against the expression levels of the corresponding controls. GUS staining (E) and GUS activity (F) of transgenic tomato transformed with *SlNAC2pro-GUS* under various saline–alkali stress. (G) Nuclear localization of SlNAC2 was detected in plant cells. SlNAC2 fused to GFP, as well as empty GFP and BES1n-mCherry (nuclear marker), was co-expressed in *N. benthamiana* leaves (50-μm scale bars). Data represent the means ± SD of three independent biological replicates. Asterisks indicate statistically significant differences (Student’s *t* test, ^**^P < 0.01, and ^***^P < 0.001) with corresponding controls.

The tomato homolog of NAC2, SlNAC2, remains largely uncharacterized, especially regarding its potential function in salt–alkali tolerance. Therefore, we focused on this gene for detailed investigation to explore the conservation and divergence of NAC2 function in tomato. Phylogenetic analysis provides crucial insights into the evolutionary relationships of a candidate protein, helping to infer its potential functions across species [12]. To perform phylogenetic analysis, protein sequences homologous to SlNAC2 were retrieved from online databases (NCBI and Sol Genomics Network), and a phylogenetic tree was subsequently constructed using the neighbor-joining (NJ) method. Sequence analysis revealed that SlNAC2, a 319-amino-acid protein, is evolutionarily conserved across plant species, sharing over 80% sequence identity with CaNAC2 (*Capsicum annuum*) and NtNAC2 (*Nicotiana tabacum*), ~60% identity with *Arabidopsis thaliana* AtNAC2, and 51% identity with rice OsNAC2 (Fig. 1A). These findings indicate that SlNAC2, a homolog of AtNAC2 and a member of the NAC transcription factor family, is presumed to be a key regulatory component in tomato’s defense mechanisms against abiotic stresses, particularly salt and alkaline stress.

To investigate the temporal and spatial expression profile of *SlNAC2* in tomato, we detected its expression across multiple tissues and organs using quantitative real-time PCR (qRT-PCR). The results showed that *SlNAC2* is ubiquitously expressed across multiple tomato tissues, with notably higher transcript accumulation observed in roots and leaves (Fig. 1B). As described above, transcriptomic studies indicate that *SlNAC2* is responsive to various abiotic stresses. To determine whether *SlNAC2* responds to salt–alkali stress, we examined its transcriptional induction under Na_2_CO_3_ condition. Consistently, Na_2_CO_3_ treatment markedly upregulated *SlNAC2* transcript levels in both roots and shoots, highlighting its induction under salt–alkali stress. Interestingly, *SlNAC2* expression peaked earlier in roots (6 h) than in shoots (12 h), indicating tissue-specific temporal dynamics in its stress-responsive regulation (Fig. 1C). Additionally, we found that *SlNAC2* expression was also induced by melatonin (MT), a bioactive molecule known to play crucial roles in plant stress tolerance (Fig. 1D). Given that MT induces *SlNAC2* expression, it is plausible that melatonin mediates SlNAC2-governed regulatory pathways in tomato adaptation to salt–alkali stress. Furthermore, in *SlNAC2pro-GUS* transgenic plants, only faint GUS staining was observed in leaves under control conditions, while the intensity of staining gradually increased with the severity of salt–alkali stress (Fig. 1E). This pattern was further corroborated by quantitative measurements of GUS enzyme activity (Fig. 1F), closely mirroring the expression pattern of *SlNAC2*. In addition, subcellular localization assays showed that the free GFP signal, serving as a control, was broadly distributed across the cell, while SlNAC2-GFP exhibited a distinct nuclear-specific signal overlapping with the nuclear marker BES1n-mCherry (Fig. 1G). Collectively, these findings suggest that SlNAC2, a nuclear-localized NAC transcription factor, functions as a key regulator in plant’s defense against saline–alkali stress, potentially through a MT-mediated signaling pathway.

### SlNAC2 acts as a negative regulator of saline–alkali stress tolerance in tomato

To investigate the function of *SlNAC2* in tomato’s response to saline–alkali stress, we utilized a VIGS-mediated strategy to downregulate *SlNAC2* expression in tomato and assessed the knockdown efficiency in *TRV-SlNAC2* transgenic lines through qRT-PCR (Fig. S1A). Compared with *TRV-control* plants, *TRV-SlNAC2* lines exhibited enhanced tolerance to saline–alkali stress, as reflected by decreased levels of physiological stress indicators, including malondialdehyde (MDA) and ion leakage, as well as increased Fv/Fm ratio and fresh weight (Fig. S2A–E). Findings from gene silencing experiments indicate that downregulation of *SlNAC2* improves tomato’s resistance to saline–alkali stress, implying that SlNAC2 may function as a negative modulator in stress signaling pathways.

**Figure 2:**
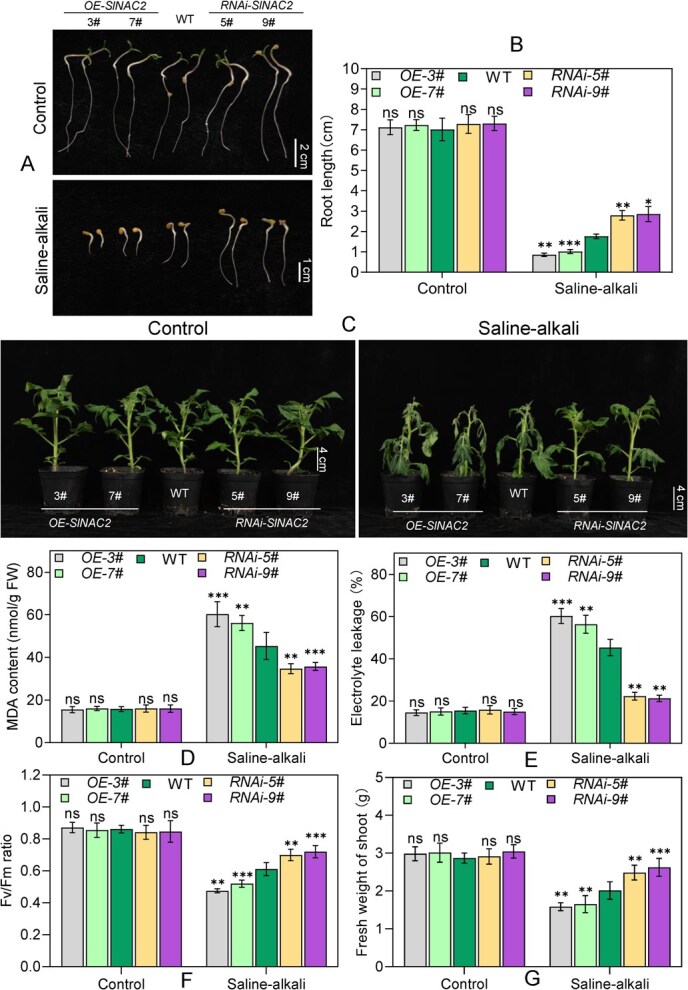
*SlNAC2* overexpression reduces saline–alkali tolerance in tomato. (A) Phenotypic analysis of root growth in transgenic and WT plants seedlings under saline–alkali stress (100 mM Na_2_CO_3_). Scale bars indicate 1 and 2 cm. (B) Statistical evaluation of root length as shown in (A). Values represent the mean of three independent biological replicates ± SD (*n* = 50). (C) Phenotypic differences between WT and transgenic plants (*OE-SlNAC2* and *RNAi-SlNAC2*) were evaluated under both normal and saline–alkali stress conditions. After 4 weeks of growth in normal conditions, plants were subjected to either no treatment (Control) or 500 mmol/l Na_2_CO_3_ for 6 days (4 cm scale bars). (D–G) MDA content (D), ion leakage (E), and Fv/Fm(F), and fresh weight (G) in (C). The values represent the mean ± SD obtained from four independent biological replicates. Asterisks indicate significant differences levels (Student’s *t* test, *P < 0.05, ^**^P < 0.01, ^***^P < 0.001; ns, not significant) with corresponding controls.

For detailed functional analysis of *SlNAC2* in stress resistance, we generated both overexpression (OE) and RNA interference (RNAi) lines in the Micro-Tom cultivar background with either constitutive overexpression or RNAi-mediated silencing of *SlNAC2*. A total of 18 *OE-SlNAC2* lines and 16 *RNAi-SlNAC2* lines were successfully generated. Among them, four representative lines *OE-3#*, *OE-7#*, *RNAi-5#*, and *RNAi-9#* were selected for subsequent characterization based on qRT-PCR expression analysis (Fig. S1B). To investigate this, we assessed the germination performance of seeds from wild-type (WT), *OE-SlNAC2*, and *RNAi-SlNAC2* tomato lines under normal and Na_2_CO₃-supplemented Hoagland solution. Under optimal conditions, no obvious phenotypic differences were observed between the transgenic lines and WT plants. However, in response to saline–alkali conditions, *RNAi-SlNAC2* tomato plants exhibited a higher germination rate than that observed in WT, whereas *SlNAC2* overexpressing lines showed a notable decrease of ~10%–15% (Fig. S3). In the absence of Na_2_CO₃, transgenic lines displayed root lengths similar to those of WT plants. Conversely, saline–alkali treatment resulted in a marked increase in root length of *RNAi-SlNAC2* plants, while a corresponding reduction was observed in *OE-SlNAC2* lines compared to WT (Fig. 2A and B). To determine saline–alkali stress response of soil-grown seedlings, transgenic and WT plants were cultivated in soil for 4 weeks, followed by a 6-day treatment with 500 mM Na_2_CO₃. Under salt–alkali conditions, seedlings overexpressing *SlNAC2* exhibited pronounced growth inhibition, whereas *RNAi* -*SlNAC2* lines showed significantly enhanced growth compared to the WT plants (Fig. 2C). In addition, compared with WT under saline–alkali stress, *RNAi*-*SlNAC2* plants displayed reduced MDA accumulation and ion leakage in leaves (Fig. 2D and E), along with higher Fv/Fm ratio and greater fresh weight (Fig. 2F and G), whereas *OE-SlNAC2* lines showed the reverse trend (Fig. 2D–G), consistent with the observed stress-related phenotypes of these transgenic plants. Based on these results, SlNAC2 can be concluded to function as a negative regulator of tomato tolerance to saline–alkali stress.

### SlNAC2 negatively modulates saline–alkali tolerance by regulating ROS and melatonin levels

Salt–alkali conditions induce oxidative stress in plants tissues by promoting the accumulation of ROS. Accordingly, we evaluated ROS levels in WT, *OE-SlNAC2*, and *RNAi*-*SlNAC2* plants through histochemical staining and absorbance spectrophotometry methods, under both control and Na_2_CO₃ stress conditions. DAB and nitro blue tetrazolium (NBT) staining, together with commercial assay kits, were employed to evaluate hydrogen peroxide (H_2_O_2_) and superoxide anion (O_2_^−^) contents, as well as related physiological parameters, in different tomato genotypes subjected to saline–alkali stress. Histochemical staining revealed that *OE-SlNAC2* plants accumulated higher levels of H_2_O_2_ and O_2_^−^ than WT, as indicated by more intense staining, while *RNAi*-*SlNAC2* lines displayed significantly weaker staining, consistent with reduced ROS (H_2_O_2_ and O_2_^−^) content (Fig. 4A and B). Quantitative analyses corroborated the histochemical staining results, showing that H_2_O_2_ and O_2_^−^ levels were significantly increased in *OE-SlNAC2* lines but decreased in *RNAi-SlNAC2* plants compared to WT (Fig. 4C and D). Moreover, *RNAi-SlNAC2* lines exhibited higher antioxidant enzyme activities (CAT, POD, APX, and SOD) than WT, whereas the overexpression lines displayed the opposite trend, consistent with the phenotypic responses of these transgenic lines to saline–alkali stress. Interestingly, as shown, melatonin levels were higher in the RNAi lines but lowest in the overexpression lines following saline–alkali stress compared with WT. These results suggest that SlNAC2 compromises tomato resistance to saline–alkali stress by regulating the ROS signaling pathway, leading to increased ROS accumulation and consequent oxidative damage.

An increasing amount of evidence demonstrates that melatonin functions as a crucial signaling molecule involved in orchestrating plant defense mechanisms under saline–alkali stress [60]. In our study, exogenous melatonin application was found to markedly elevate the transcript levels of *SlNAC2*, implying an interaction between melatonin-mediated signaling and SlNAC2-dependent stress adaptation. Consequently, we quantified endogenous melatonin concentrations in transgenic lines and their corresponding WT controls. As shown in Fig. 4I, melatonin levels were comparable among the tested genotypes under nonstress conditions, with no significant differences observed. When subjected to salt–alkali treatment, the levels of endogenous melatonin were notably elevated in *RNAi*-*SlNAC2* plants, while those overexpressing *SlNAC2* lines showed a significant decline in melatonin synthesis relative to WT controls. Taken together, these findings indicate that SlNAC2 exerts a negative effect in tomato tolerance to saline–alkali stress, potentially by promoting ROS accumulation and disrupting melatonin-mediated scavenging mechanisms.

Earlier experiments demonstrated that the increased saline–alkali stress susceptibility was observed in overexpressing *SlNAC2* plants, which was associated with a marked reduction in melatonin accumulation relative to WT. To test the hypothesis that melatonin deficiency is responsible for the salt–alkali hypersensitivity in *OE-SlNAC2* plants, we treated the plants with exogenous melatonin and compared their responses to those of WT controls. Compared to plants subjected to salt–alkali stress without melatonin application, melatonin pretreatment significantly mitigated the adverse effects of saline–alkali stress on plant growth over the 6-day treatment period (Fig. S4A). Melatonin pretreatment substantially enhanced physiological performance, as evidenced by increased fresh biomass (Fig. S4B). Moreover, levels of oxidative stress indicators, such as ion leakage and MDA, were markedly reduced compared to those in stressed plants without melatonin application (Fig. S5C and D). Meanwhile, we analyzed the effects of melatonin on plant growth under nonstress conditions. No significant differences were observed between MT-treated and untreated wild-type or *OE-SlNAC2* plants. These data suggest that the effects of MT observed under salt–alkali stress are not attributable to growth promotion under normal conditions (Fig. S5A–C). Together, it can be inferred from these results that melatonin may function as a downstream effector within SlNAC2-dependent regulatory network, orchestrating adaptive response to saline–alkali stress in tomato.

### Characterization of downstream targets of SlNAC2 in tomato under saline–alkali stress

Given that *OE-SlNAC2* plants exhibit altered responses to salt stress, we next investigated which downstream effectors might be responsible by measuring the transcript levels of several well-documented salt-responsive genes, including *SlSOS1*, *SlSOS2*, *SlCBL10*, *SlHKT1;1*, and *SlDREB2* [17, 64], in *OE-SlNAC2*, WT, and *RNAi-SlNAC2* plants under normal and saline–alkali stress conditions using qRT-PCR. As shown in the results (Fig. 4A–E), when grown under control conditions, transgenic and WT plants exhibited similar transcript abundances of the examined genes. Notably, knockdown of *SlNAC2* triggered pronounced increases in *SlSOS1*, *SlHKT1;1*, and *SlDREB2* expression, whereas these genes were significantly repressed in *OE-SlNAC2* plants relative to WT. It is noteworthy that *SlSOS2* and *SlCBL10* transcript levels did not differ between transgenic and WT plants. It has recently been reported that, through the regulation of ROS homeostasis, SlCV serves as an essential downstream component by which melatonin influences leaf senescence in tomato. Therefore, we examined whether *SlCV* transcript levels are altered in WT and *SlNAC2* transgenic lines under saline–alkali conditions. The qRT-PCR data showed that *SlNAC2* overexpression resulted in strong induction of *SlCV*, while silencing *SlNAC2* led to its significant down-regulation compared with WT (Fig. 4F). In addition, we also found that *SlCV* transcript levels were significantly elevated in response to saline–alkali stress (Fig. S8A). Previous studies have shown that SlNAC12 positively regulates salt tolerance in tomato [65]. Thus, we performed quantitative expression analyses to examine the potential relationship between SlNAC2 and SlNAC12. Our results show that the expression level of *SlNAC12* is not directly correlated with SlNAC2 in *OE-SlNAC2* plants, and similarly, *SlNAC2* expression is not directly affected in *OE-SlNAC12* plants (Fig. S8E and F). These results suggest that SlNAC2 and SlNAC12 may regulate saline–alkali stress responses through largely independent pathways. Overall, these findings demonstrate that SlNAC2 modulates tomato salt–alkali tolerance largely through its regulation of those key stress-responsive genes.

Considering the findings described above on melatonin, we hypothesized that SlNAC2 modulates tomato adaptation to saline–alkali stress primarily by fine-tuning the melatonin biosynthetic pathway. Thereby, we examined the transcript levels of a myriad of previously characterized key genes involved in melatonin biosynthesis, including *SlCOMT1*, *SlCOMT2*, *SlT5H*, *SlAMST5*, *SlAMST7*, and *SlSNAT,* in WT and transgenic plants through qRT-PCR under control and saline–alkali stress conditions [54, 63]. In the absence of salt–alkali stress, no apparent alteration in the expression levels of these target genes were detected between WT and *OE-SlNAC2* or *RNAi- SlNAC2* lines. Compared to WT plants, the transcriptional levels of *SlCOMT1*, *SlCOMT2*, and *SlSNAT* were markedly reduced in *OE-SlNAC2* lines, whereas a notable upregulation of these genes was detected in *RNAi- SlNAC2* plants in response to salt–alkali stress (Fig. 4G–I). Notably, saline–alkali treatment did not cause significant changes in the expression of *SlT5H*, *SlAMST5*, and *SlAMST7* across the genotypes tested (Fig. S11A–C). These collective observations suggest that SlNAC2 modulates tomato tolerance to saline–alkali stress by regulating the transcription of multiple genes responsible for melatonin biosynthesis, thereby implicating a potential mechanistic pathway underlying salt and alkali stress response.

### SlNAC2 exerts transcriptional regulation of *SlCOMT2*, *SlSNAT*, and *SlCV* through direct promoter binding during saline–alkali stress

Evidence from our study demonstrates that the transcript levels of *SlCOMT1*, *SlCOMT2*, *SlSNAT*, and *SlCV* are under the regulatory control of SlNAC2 during saline–alkali stress. To elucidate the role of SlNAC2 in plant adaptation to saline–alkali stress at the transcriptional level, we examined its direct regulatory effects on the expression of the aforementioned genes. Promoter analysis through the PlantTFDB database revealed that all four candidate genes harbor predicted TAGC elements, which are putative binding sites for NAC transcription factors (Fig. 5A). ChIP-qPCR analysis was performed to validate *in vivo* binding of SlNAC2 to the promoters of these target genes, revealing significant enrichment of SlNAC2 at promoter regions of *SlCOMT2*, *SlSNAT*, and *SlCV* (Fig. 5A). To further evaluate the potential capacity of SlNAC2 to target the promoter regions of *SlCOMT2*, *SlSNAT*, and *SlCV*, yeast one-hybrid (Y1H) assays were conducted. Constructs containing *SlCOMT2*, *SlSNAT*, and *SlCV* promoter fragments were inserted into the pHIS2.1 reporter vector, while SlNAC2 was cloned into the vector pGADT7; the resulting plasmids were co-transformed into Y1HGold yeast cells for interaction testing. Our findings from Y1H assays reveal that SlNAC2 directly interacts with the promoter regions of *SlCOMT2*, *SlSNAT*, and *SlCV* (Fig. 5B)*.* To gain deeper insights into the interaction mentioned above, a dual-luciferase reporter assay was carried out in tobacco leaves, using constructs containing *SlCOMT2*, *SlSNAT*, and *SlCV* promoters in *pGreenII0800-LUC* and *SlNAC2* CDS in *pGreenII62-SK-GFP*. In the dual-luciferase assay, co-expression of *SlNAC2* with either the *SlCOMT2pro*-LUC or *SlSNATpro*-LUC reporters construct significantly reduced the LUC/REN ratio compared to the control group, indicating that SlNAC2 exerts a repressive effect on promoter activity. In contrast, when *SlNAC2* was co-expressed with the *SlCVpro-LUC* construct, a pronounced increase in the LUC/REN ratio was observed, demonstrating that SlNAC2 acts as a transcriptional activator of *SlCV* (Fig. 5C). An electrophoretic mobility shift assay (EMSA) experiment was subsequently performed to investigate whether SlNAC2 could bind specifically to cis-elements within the promoters, which demonstrated that the His-tagged SlNAC2 recombinant protein strongly interacted with these sequences. Importantly, competition assays revealed that only the unlabeled wild-type probes, but not the mutated variants, effectively competed with labeled probes for SlNAC2 binding (Fig. 5D–F). Protein–DNA binding assays (EMSA) confirmed the physical interaction of SlNAC2 with the promoter fragments of *SlCOMT2*, *SlSNAT*, and *SlCV in vitro*. Overall, these results demonstrate that SlNAC2 directly binds to the promoters of *SlCOMT2*, *SlSNAT*, and *SlCV*, repressing *SlCOMT2* and *SlSNAT* to lower endogenous melatonin levels, while activating *SlCV* to enhance ROS accumulation, thereby increasing tomato susceptibility to salt–alkali stress.

### Knockdown of *SlCOMT2* in *RNAi-SlNAC2* plants partially compromises their enhanced tolerance to saline–alkali stress

Given their direct transcriptional regulation by SlNAC2, SlCOMT2, a key enzyme in the melatonin biosynthetic pathway, is likely to play crucial roles in modulating tomato tolerance to saline–alkali stress. To further substantiate the role of SlNAC2 in modulating tomato salt stress response via SlCOMT2, we employed VIGS to suppress *SlCOMT2* expression in *RNAi*-*SlNAC2* plants, generating the *RNAi*-*SlNAC2/TRV-SlCOMT2* and *SlNAC2-RNAi/TRV-*Control lines. Compared to the control lines, *SlNAC2-RNAi/TRV-SlCOMT2* showed pronounced susceptibility and aggravated wilting symptoms upon saline–alkali treatment (Fig. 6A). Consistent with the stress-sensitive phenotype, *RNAi*-*SlNAC2/TRV-SlCOMT2* lines displayed elevated oxidative stress and membrane damage, as indicated by increased levels of ion leakage, coupled with suppressed fresh weight and diminished melatonin content in comparison to controls, following saline–alkali treatment (Fig. 6B–E). In contrast, plants overexpressing *SlCOMT2* displayed enhanced tolerance under the same stress conditions (Fig. S6). Based on the observed genetic interactions, SlCOMT2 functions downstream of SlNAC2 within the regulatory cascade mediating salt and alkali stress responses. In conclusion, these results suggest that SlCOMT2, as a target of SlNAC2, is repressed by SlNAC2, indicating that modulation of melatonin homeostasis contributes to the fine-tuning of tomato salt–alkali stress responses.

### SlDREB2 physically interacts with SlNAC2 to modulate its transcriptional activity

A myriad of investigations indicates that DREB can physically interact with NAC transcription factors, thereby modulating plant responses to salinity [41, 45, 50]. Additionally, studies in tomato have shown that *SlDREB1*, *SlDREB2*, and *SlDREB3* play important roles in regulating responses to various abiotic stresses [45, 50, 66]. Given the preceding findings, we aimed to investigate the expression patterns of *SlDREB1*, *SlDREB2*, and *SlDREB3* under saline–alkali stress. The qRT-PCR analysis revealed that the expression levels of *SlDREB2* and *SlDREB3* were markedly induced by saline–alkali stress, reaching more than 10-fold higher than those in the control, whereas *SlDREB1* showed no significant change (Fig. S8B–D). The result suggests that *SlDREB2* and *SlDREB3* may play important roles in the response of tomato to saline–alkali stress. Integrating our findings with previous reports, we propose that SlDREB2 and/or SlDREB3 may functionally interact with SlNAC2 to orchestrate a precise regulatory network governing tomato tolerance to saline–alkali stress. To validate the proposed interaction, bimolecular fluorescence complementation (BiFC) assays were performed to examine the formation of SlDREB2–SlNAC2 and SlDREB3–SlNAC2 protein complexes. Strong YFP fluorescence signals were observed in *Nicotiana benthamiana* leaves co-expressing SlNAC2-nYFP and SlDREB2-cYFP, which colocalized with the nuclear marker BES1n-mCherry. In contrast, no YFP fluorescence was detected in the combination of SlDREB3 and SlNAC2 constructs, indicating that only SlDREB2 interacts with SlNAC2 *in vivo*(Fig. 7A and Fig. S10). To further verify the interaction between SlDREB2 and SlNAC2, a yeast two-hybrid (Y2H) assay was performed by co-transforming both constructs into yeast cells. The results demonstrated that only cells expressing both SlDREB2 and SlNAC2 were able to grow on selective medium, whereas single transformants failed to do so, indicating a specific protein–protein interaction (Fig. 7B). Supporting the interaction, Co-IP results indicated that GFP-SlNAC2 could co-immunoprecipitate 6xMYC-SlDREB2, thereby confirming their direct binding (Fig. 7C).

Since SlNAC2 directly targets the promoters of *SlCOMT2* and *SlSNAT* and physically interacts with SlDREB2, we hypothesized that SlDREB2 may also directly bind to these promoters, potentially contributing to cooperative transcriptional regulation. To verify this hypothesis, Y1H assays were conducted. As shown in the Fig. S9, yeast cells co-transformed with SlDREB2 and the *SlCOMT2* or *SlSNAT* promoter constructs failed to grow on selective triple-dropout medium, whereas the positive control (SlNAC2 + *SlCOMT2* promoter) exhibited normal growth. These results indicate that SlDREB2 does not directly bind to the promoters of *SlCOMT2* and *SlSNAT*. Furthermore, to assess the impact of SlDREB2 on SlNAC2-mediated transcriptional regulation, we conducted a dual-luciferase assay in which *pGreenII62-SK-SlNAC2* was introduced into tobacco leaves either independently or together with pGreenII62-SK-SlDREB2, along with reporter constructs *pGreenII0800-SlCOMT2pro-LUC*, *pGreenII0800-SlSNATpro-LUC*, and *pGreenII0800-SlCVpro-LUC*. Experimental evidence suggests that SlDREB2 mitigates the transcriptional inhibition of SlNAC2 on its targets *SlCOMT2* and *SlSNAT*, while also attenuating SlNAC2-mediated activation of *SlCV* transcription (Fig. 7D). To investigate the functional interplay between SlNAC2 and SlDREB2, *SlNAC2-OE/TRV-SlDREB2* and corresponding *TRV-Control* lines in the genetic background of *SlNAC2-OE* were generated by VIGS-mediated silencing of *SlDREB2*. Saline–alkali exposure triggered a pronounced wilting response in *SlNAC2-OE/TRV-SlDREB2* lines (Fig. 8A), indicating increased stress susceptibility relative to control plants. Simultaneously, stress-induced biochemical analyses revealed that the loss of functional *SlDREB2* rendered *SlNAC2-OE* plants more susceptible to saline–alkali stress, as evidenced by markedly elevated levels of MDA and ion leakage, coupled with a significant decline in fresh weight and Fv/Fm ratio, compared with their control counterparts (Fig. 8B–E). In parallel, we generated stable *SlDREB2*-overexpressing lines and evaluated their responses to saline–alkali stress. Notably, *OE-SlDREB2* plants displayed significantly enhanced tolerance relative to wild-type plants. Together, these genetic interaction analyses provide strong evidence that SlDREB2 acts in concert with SlNAC2 to regulate tomato salt–alkali stress tolerance (Fig. S7).

In summary, these results demonstrate that SlNAC2 physically interacts with SlDREB2 to form a functional transcriptional complex that exerts precise and fine-tuned control over downstream genes governing melatonin biosynthesis and ROS homeostasis, thereby ensuring an optimal physiological response of tomato to saline–alkali stress.

## Discussion

### SlNAC2 functions as a negative regulator of tomato adaptation to saline–alkali stress

The widespread occurrence of soil salinization, affecting ~20% of irrigated farmland worldwide, presents a formidable obstacle to agricultural productivity and food security [9, 10]. Due to their moderate salt sensitivity, tomato plants undergo growth retardation and developmental impairment under high salt and alkali conditions, with possible consequences including severe yield reduction or plant mortality. In response to saline–alkali conditions, plants trigger multiple protective mechanisms to preserve cellular integrity and support sustained development under environmental stress. Transcription factors establish a regulatory network that finely tunes the expression of genes essential for stress tolerance [67, 68]. Considerable research has focused on several important families of transcription factors, notably NAC, DREB, WRKY, and bHLH [17, 41, 69, 70]. Extensive evidence highlights the involvement of NAC transcription factors in plant responses to salt and alkali stress [20]. Nonetheless, the molecular mechanisms by which NAC factors confer stress tolerance in tomato remain largely unknown, and deciphering these pathways could inform the development of stress-resilient tomato cultivars.

**Figure 3:**
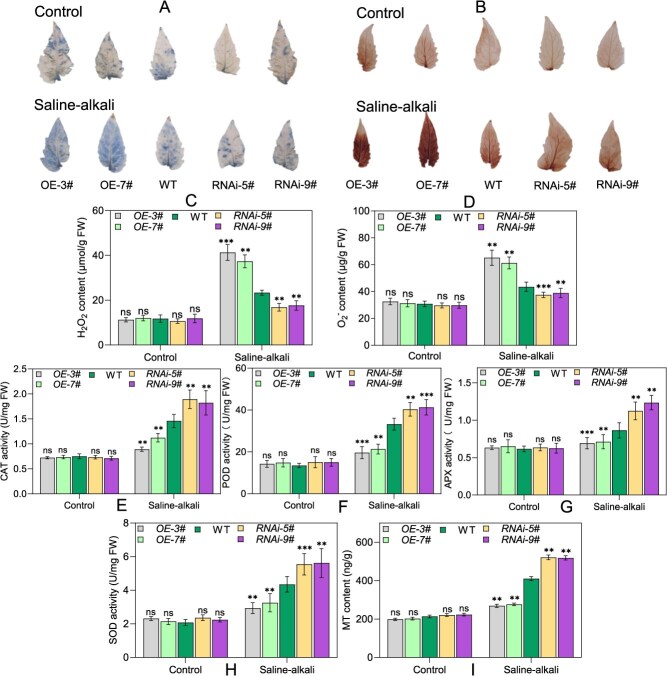
*SlNAC2* negatively regulates ROS detoxification and MT accumulation under saline–alkali stress. (A) NBT staining. (B) DAB staining. (C) H_2_O_2_ content. (D) O_2_^−^ content. (E) CAT activity. (F) POD activity. (G) APX activity. (H) SOD activity. (I) MT content. Data represent the means ± SD of three independent biological replicates. Physiological indices were measured in the leaves of WT and transgenic plants following 5 days of 0 or 500 mM Na_2_CO_3_ treatment at the 4-week growth stage. Asterisks indicate statistically significant differences (^**^P < 0.01, ^***^P < 0.001 by the Student’s test; ns, not significant) from the corresponding control.

Extensive evidence from rice indicates that OsNAC2 orchestrates a range of developmental and stress-related processes [21–23, 31–33, 71], such as plant height regulation, leaf senescence, root development, and disease and abiotic stress responses, highlighting its role as a pivotal regulatory hub. The functional characterization of SlNAC2 in tomato, however, remains largely unexplored. Investigating SlNAC2 may therefore yield critical insights into tomato development and stress tolerance, and inform future crop improvement strategies. Previous transcriptome analyses have shown that SlNAC2 is significantly upregulated under environmental stress conditions [72–74], suggesting a potential regulatory role in tomato stress adaptation. Based on this, we hypothesized its involvement in stress signaling and conducted a detailed functional characterization in this study. This work presents the identification and functional characterization of SlNAC2, a nuclear-localized NAC transcription factor from tomato that plays a role in the plant’s response to saline–alkali stress. Under saline–alkali conditions, the expression of *SlNAC2* was significantly upregulated, during which the protein acted as a negative regulator of transcription. In addition, GUS histochemical analyses of *SlNAC2pro-GUS* transgenic tomato plants indicated low basal expression in leaves under nonstress conditions, which was greatly intensified following saline–alkali treatment, highlighting the stress-inducible nature of *SlNAC2* expression (Fig. 1). Stress assays revealed that tomato plants overexpressing *SlNAC2* exhibited heightened vulnerability to salt–alkali stress, in contrast to *RNAi-SlNAC2* lines, which showed significantly enhanced tolerance to saline–alkali stress (Figs 2 and 3). Collectively, our findings demonstrate that SlNAC2 acts as a negative modulator of saline–alkali stress tolerance in tomato. Moreover, as shown in Fig. 1D, melatonin (MT) treatment markedly increased the transcript abundance of *SlNAC2*, suggesting that this gene may participate in the plant’s MT-responsive mechanisms. Consequently, a more detailed investigation into the role of SlNAC2 in MT-associated signaling pathways is essential for a comprehensive understanding of its regulatory function.

**Figure 4:**
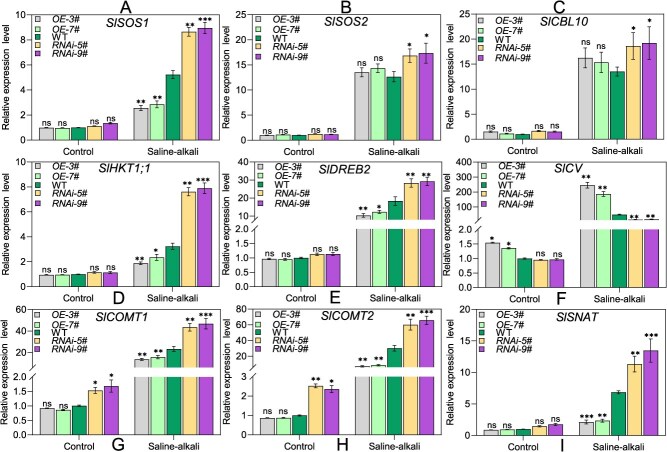
Transcript profiles of stress-associated genes and MT biosynthetic genes in WT and transgenic plants during salt–alkali stress. The relative expression levels of *SlSOS1* (A), *SlSOS2* (B), *SlCBL10* (C), *SlHKT1;1* (D), *SlDREB2* (E), *SlCV* (F), *SlCOMT1* (G), *SlCOMT2* (H), and *SlSNAT* (I) in *OE-SlNAC2*, *RNAi-SlNAC2*, and WT plants. Following 12 h of treatment with either control (0 mM) or 200 mM Na_2_CO_3_, plants were harvested for RNA extraction and gene expression analysis. *SlActin2* was utilized as the internal reference gene. The values represent the mean ± SD obtained from three independent biological replicates. Asterisks indicate significant differences levels (Student’s *t* test, ^*^P < 0.05, ^**^P < 0.01, ^***^P < 0.001; ns, not significant) with corresponding controls.

**Figure 5:**
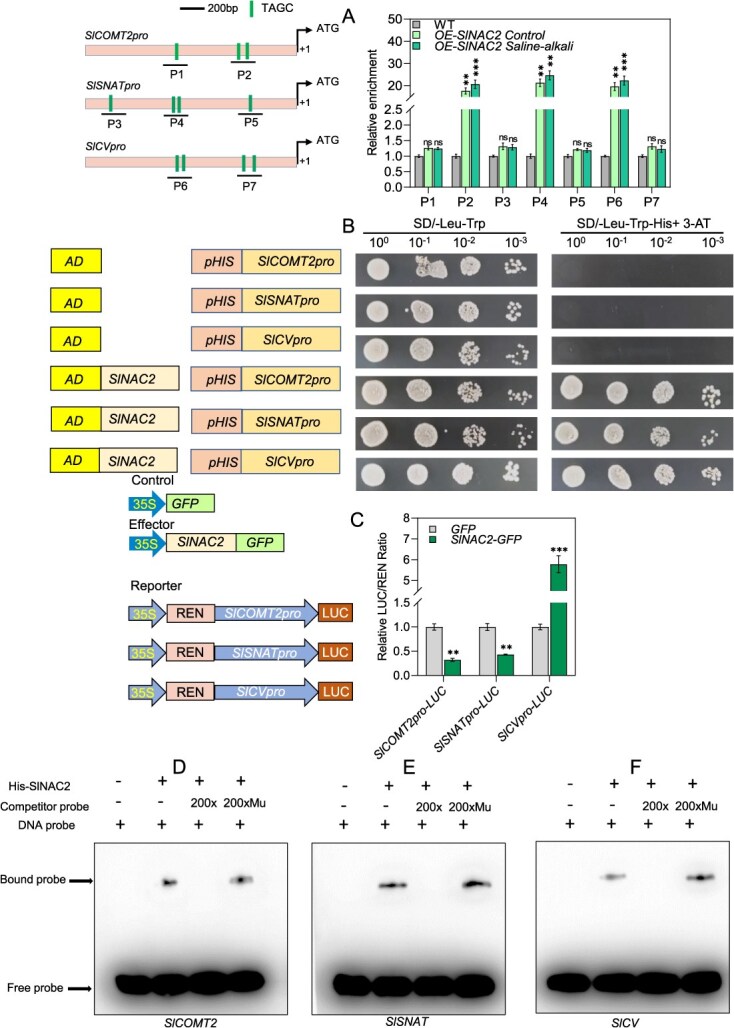
SlNAC2 directly binds to the promoters of *SlCOMT2*, *SlSNAT*, and *SlCV* to regulate their expression. (A) Schematic representations of the *SlCOMT2*, *SlSNAT*, and *SlCV* promoters, with TAGC elements highlighted in green rectangles. The ChIP-qPCR analysis demonstrates that SlNAC2 directly interacts with the promoters of *SlCOMT2*, *SlSNAT*, and *SlCV*. DNA immunoprecipitation was performed using an anti-GFP monoclonal antibody on 4-week-old *35S:SlNAC2-GFP* transgenic tomato. Data represent the means ± SD of four independent biological replicates. Asterisks indicate statistically significant differences (^**^P < 0.01, ^***^P < 0.001 by the Student’s test) from the corresponding control. (B) In yeast cells, SlNAC2 associates with the promoter regions of *SlCOMT2*, *SlSNAT*, and *SlCV*. (C) Dual-LUC assays demonstrate that SlNAC2 suppresses the promoter activities of *SlCOMT2* and *SlSNAT* while activating *SlCV*. (D-F) EMSA analysis reveals direct binding of His-SlNAC2 to the promoter regions of *SlCOMT2*, *SlSNAT*, and *SlCV in vitro*. For competition assays, unlabeled or mutated probes were employed.

**Figure 6:**
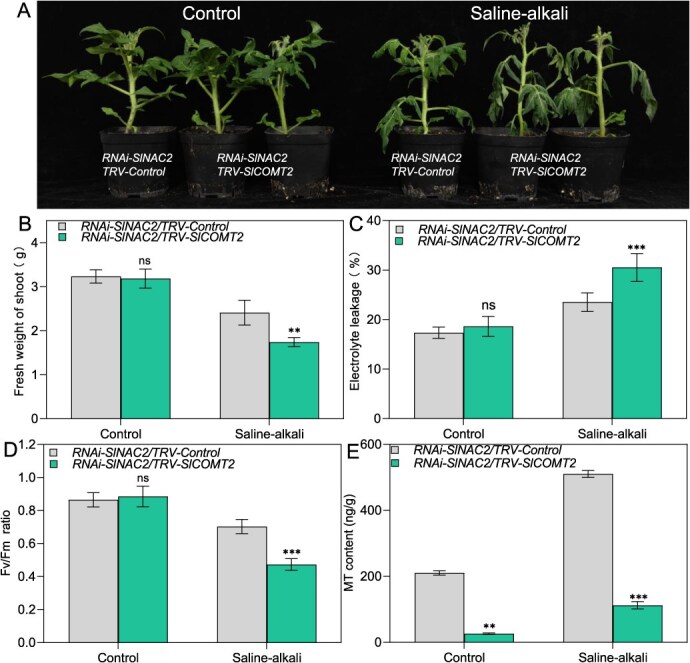
*SlCOMT2* serves as a downstream effector of SlNAC2 in regulating salt–alkali tolerance. (A) Growth phenotypes of *RNAi-SlNAC2/Control* and *RNAi-SlNAC2/TRV-SlCOMT2* plants in the absence or presence of saline–alkali stress (bar = 4 cm). Plants grown for 4 weeks under normal conditions were exposed to control or 500 mM Na_2_CO_3_ treatment for 6 days. Fresh weight (B), ion leakage (C), Fv/Fm (D), MT content (E). Data represent the means ± SD of four independent biological replicates. Asterisks indicate statistically significant differences (^**^P < 0.01, ^***^P < 0.001 by the Student’s test; ns, not significant) from the corresponding control.

**Figure 7:**
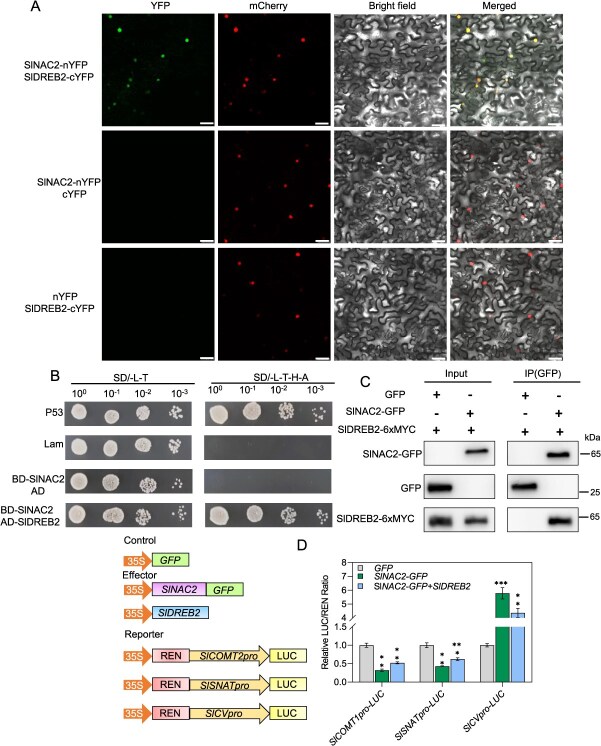
SlDREB2 physically associates with SlNAC2 and modulates its transcriptional regulatory activity. (A) BiFC assays showed that SlDREB2 physically interacts with SlNAC2 in cells, and the fluorescence signals overlap with BES1n-mCherry (a nuclear marker). (B) Y2H analysis showed that SlDREB2 physically interacted with SlNAC2 in yeast cells. (C) Co-IP assays demonstrated the *in vivo* interaction between SlDREB2 and SlNAC2. Immunoprecipitation of total proteins was performed using anti-GFP trap magnetic agarose beads, and the co-immunoprecipitated SlDREB2-6xMYC was identified using an anti-MYC antibody. Three independent experiments produced comparable results. (D) The results from Dual-LUC assays showed that SlDREB2 physically interacts with SlNAC2, thereby influencing its transcriptional activity. Data represent the means ± SD of three independent biological replicates. Asterisks indicate statistically significant differences (^**^P < 0.01, ^***^P < 0.001 by the Student’s test; ns, not significant) from the corresponding control.

**Figure 8:**
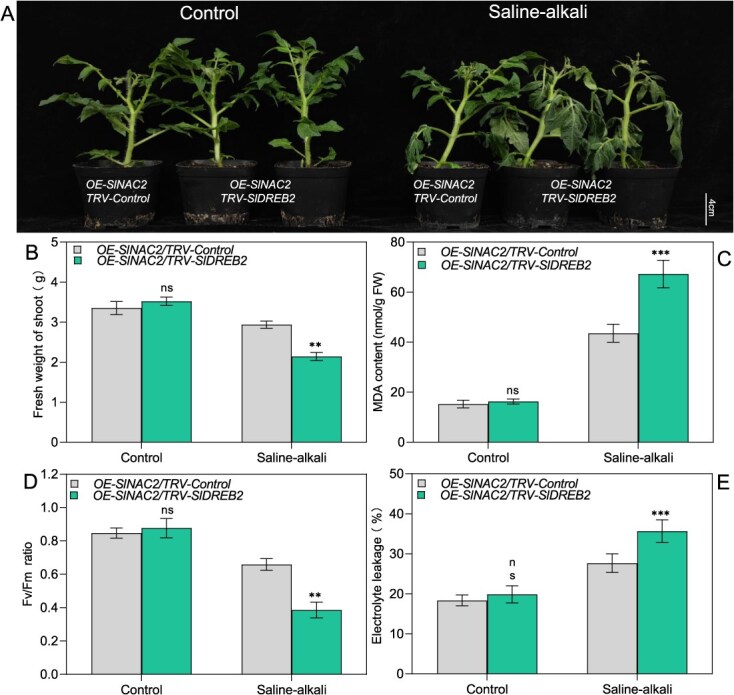
SlDREB2 knockdown enhances the sensitivity of *SlNAC2*-overexpressing plants to salt–alkali stress. (A) Phenotypic comparison of *OE-SlNAC2/Control* and *OE-SlNAC2/TRV-SlDREB2* plants under control and salt–alkali stress conditions. Stress-related physiological parameters measured in these transgenic plants (A), fresh weight (B), MDA content (C), Fv/Fm (D), ion leakage (E). Data represent the means ± SD of three independent biological replicates. Asterisks indicate statistically significant differences (^**^P < 0.01, ^***^  *P* < 0.001 by the Student’s test; ns, not significant) from the corresponding control.

**Figure 9:**
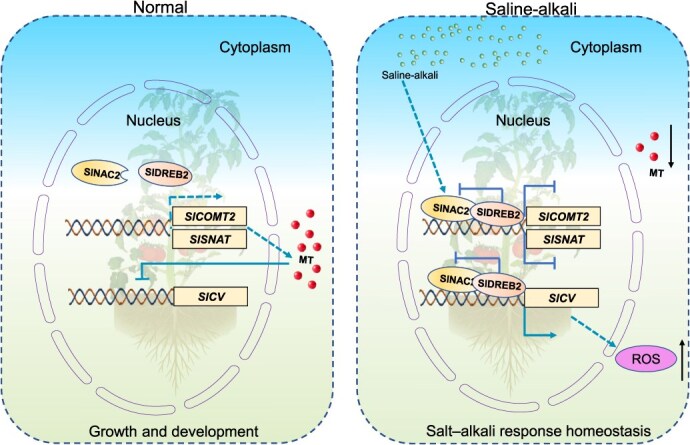
Schematic model depicting the role of SlNAC2 in regulating saline–alkali stress tolerance in tomato. Following exposure to saline–alkali stress, SlNAC2 is upregulated and directly targets the *SlCV* promoter, leading to transcriptional activation of *SlCV* and increased ROS levels. In addition, SlNAC2 interacts with the promoters of *SlCOMT2* and *SlSNAT*, resulting in transcriptional repression and a subsequent decline in endogenous MT content. The reduction in MT further enhances tomato sensitivity to salt–alkali stress. Furthermore, SlDREB2 physically interacts with SlNAC2 and modulates its transcriptional activity, reinforcing that the SlDREB2- SlNAC2 module acts as a central hub controlling saline–alkali stress responses by precisely regulating downstream genes involved in MT biosynthesis and ROS homeostasis.

Previous study has demonstrated that SlNAC12 functions as a positive regulator of salt tolerance in tomato [65]. Although both SlNAC12 and SlNAC2 belong to the NAC transcription factor family, their regulatory roles in saline–alkali stress responses appear to be distinct. Our quantitative expression analyses revealed no direct correlation between *SlNAC2* and *SlNAC12* expression levels: *SlNAC12* expression was not significantly altered in *OE-SlNAC2* plants, and conversely, *SlNAC2* expression remained unchanged in OE-SlNAC12 plants. These results suggest that SlNAC2 and SlNAC12 may act through largely independent regulatory pathways during saline–alkali stress responses. Furthermore, although SlNAC12 has been reported to regulate salt tolerance through specific downstream targets, the regulatory network associated with SlNAC2 remains largely unexplored. The apparent independence of SlNAC2 from SlNAC12 regulation highlights the potential uniqueness of SlNAC2 in mediating saline–alkali stress tolerance in tomato(Fig. S8E and F). Further studies will be required to elucidate the downstream regulatory network and interacting proteins associated with SlNAC2.

Interestingly, recent studies have shown that overexpression of *SlNAC2* in *Arabidopsis* confers enhanced tolerance to multiple abiotic stresses, which is opposite to the phenotype of SlNAC2-overexpressing tomato plants [75]. We hypothesize that this discrepancy may arise from species-specific regulatory frameworks, differences in interacting proteins, or distinct transcriptional landscapes that shape SlNAC2-dependent responses. SlNAC2 acts as a repressive regulator of tomato tolerance to saline–alkali stress. Uncovering such repressive regulators is critical for delineating the dynamic equilibrium of plant stress signaling networks, since negative elements play indispensable roles in fine-tuning or limiting defense overactivation, thereby maintaining an optimal balance between stress tolerance and growth.

### SlNAC2 represses *SlCOMT2* and *SlSNAT* to reduce melatonin while activating *SlCV* to promote ROS accumulation under saline–alkali stress

As a multifunctional hormone in plants, melatonin is deeply involved in regulating development, secondary metabolite production, and responses to environmental stresses [76]. A growing body of research suggests that melatonin plays a crucial protective role in plants under abiotic stress, especially when its intracellular levels are precisely regulated within an optimal range [77]. In response to environmental stress, melatonin strengthens the plant’s defense against stress-induced oxidative damage by boosting antioxidant enzyme systems and facilitating the accumulation of nonenzymatic antioxidants [59, 78]. In this study, experimental results demonstrated that the transcript levels of *SlNAC2* were significantly upregulated under salt–alkali conditions, and that exogenous melatonin application enhances tomato tolerance to these conditions, suggesting a functional interplay between melatonin signaling and SlNAC2-mediated stress responses. Quantitative analysis of endogenous melatonin revealed no significant differences between transgenic lines and WT controls under normal conditions. Under saline–alkali stress, however, melatonin levels were substantially decreased in *SlNAC2*-overexpressing lines, whereas RNAi lines showed elevated melatonin relative to WT. These observations support that SlNAC2 diminishes tomato resilience to saline–alkali stress, likely through interfering with melatonin-dependent protective scavenging systems. Melatonin biosynthesis in plants occurs through mitochondrial and chloroplast-localized pathways and involves several key enzymes, including TDC, T5H, SNATs, AMSTs, and COMT [51]. In this research, to explore the regulatory influence of SlNAC2 on pivotal genes involved in melatonin biosynthesis (*SlCOMT1*, *SlCOMT2*, *SlAMST5*, *SlAMST7*, and *SlSNAT*) under saline–alkali stress, we conducted Dual-LUC reporter assays and ChIP-qPCR analyses. SlNAC2 directly binds to the promoters of *SlCOMT2*, *SlSNAT*, and *SlCV*, repressing *SlCOMT2* and *SlSNAT* to lower endogenous melatonin levels, while activating *SlCV* to enhance ROS accumulation, thereby increasing tomato susceptibility to salt–alkali stress. Recent studies have highlighted the pivotal roles of *SlCOMT2* in mediating salt–alkali stress tolerance and in shaping fruit quality traits in tomato [54, 60]. Based on these findings, we selected *SlCOMT2* for further investigation to dissect its genetic relationship with SlNAC2. In this study, we demonstrated that *OE-SlCOMT2* plants displayed enhanced tolerance under saline–alkali stress. Furthermore, following salt exposure, the *RNAi-SlNAC2/TRV-SlCOMT2* plants exhibited more severe wilting and greater stress sensitivity than the corresponding control lines. Through integrated genetic and phenotypic analyses, our results support a functional linkage between SlNAC2 and SlCOMT2, indicating that SlCOMT2 acts as an important downstream component of the SlNAC2 regulatory pathway. Specifically, SlNAC2 directly regulates SlCOMT2 expression, thereby modulating melatonin biosynthesis. Given the well-established role of melatonin in plant stress adaptation, these findings suggest that SlNAC2 compromises saline–alkali stress tolerance, at least in part, by controlling melatonin production via a SlCOMT2-dependent mechanism. Collectively, our study defines a SlNAC2–SlCOMT2 regulatory module that integrates stress-responsive signaling with developmental regulation in tomato.

In *Arabidopsis*, silencing of *AtCV* has been shown to enhance tolerance to water deficit by preserving chloroplast integrity. In rice, *OsCV*-related genes are induced under drought stress, and OsCV functions as a scaffold that recruits peroxisomal proteins while facilitating the turnover of chloroplast proteins [79, 80]. More recently, evidence from tomato revealed that SlCV, through the regulation of ROS homeostasis, acts as an essential downstream component by which melatonin influences leaf senescence [56]. Consistent with these observations, our study further demonstrated that *SlCV* expression is markedly induced under saline–alkali stress (Fig. S8A), and this induction is strongly enhanced in *SlNAC2*-overexpressing tomato lines, where SlNAC2 directly binds to the *SlCV* promoter to activate its transcription (Figs 4, 5, and 7). Collectively, these findings highlight the pivotal role of SlCV in coordinating stress responses across different plant species. Importantly, they also suggest that *CV* genes may function as conserved hubs integrating diverse stress signals into ROS-mediated pathways, thereby offering potential targets for improving crop resilience to multiple abiotic stresses. Future research should explore whether CV-mediated regulatory modules contribute to cross-talk among drought, salinity, and alkaline stress pathways, which could open new avenues for developing broad-spectrum stress-tolerant crops.

### SlDREB2 interacts with SlNAC2 to form a heterodimeric complex that modulates SlNAC2’s regulatory effect on *SlCOMT2, SlSNAT,* and *SlCV* expression

Defined by a conserved AP2/ERF DNA-binding domain, DREB transcription factors are integral to plant abiotic stress signaling [81, 82]. DREB1s predominantly regulate cold responses, while DREB2s function in heat, drought, and salinity tolerance, although several DREB2 members display broader roles across multiple stress conditions [39, 48, 50, 66, 83]. Although the tomato genome encodes ~60 members of the DREB subfamily [82], the biological functions of most DREB transcription factors remain poorly characterized. Only a limited number have been characterized, such as SlDREB1, which modulates drought tolerance [84], SlDREB2 in salt stress resistance [50], and SlDREB3 in chilling tolerance [66]. Additionally, previous studies also suggest that DREB and NAC proteins work cooperatively to regulate the mechanisms governing plant salt tolerance. For example, spruce PwNAC11 forms protein complexes with *Arabidopsis* DREB2A and ABF3, contributing to the modulation of osmotic stress resistance [85]. Moreover, CmoDREB2A from pumpkin associates with CmoNAC1 and plays a positive regulatory role in improving salinity stress resilience in grafted cucumber plants [41]. Collectively, existing evidence supports the possibility that NAC–DREB transcriptional complexes function in tomato to modulate stress-adaptive responses. This research demonstrated, through Y2H assays, that SlDREB2 is a saline–alkali stress-responsive transcription factor that physically interacts with SlNAC2. The interaction between the proteins was substantiated via both BiFC and Co-IP methods (Fig. 7). Despite the identification of SlNAC2–DREB2 interaction, whether other DREB transcription factors are involved remains unclear. Furthermore, uncovering interacting proteins that modulate SlNAC2 post-translationally, including ubiquitin ligases and protein kinases, will be essential for a comprehensive understanding of its regulatory mechanism.

This study further investigated whether the SlNAC2–SlDREB2 protein complex modulates SlNAC2-mediated transcriptional regulation of downstream genes. First, Y1H assays were performed to demonstrate that SlDREB2 does not directly bind to the *SlCOMT2* or *SlSNAT* promoter regions. Luciferase activity measurements demonstrated that SlDREB2 counteracts the repressive transcriptional activity of SlNAC2 on *SlCOMT2* and *SlSNAT*, while also attenuating SlNAC2-mediated activation of *SlCV* transcription. While SlDREB2 is incapable of directly associating with the *SlCOMT2* or *SlSNAT* promoter regions, its interaction with SlNAC2 implies a co-regulatory role, whereby SlDREB2 fine-tunes SlNAC2-mediated transcriptional regulation. Such interactions between DNA-binding and non-DNA-binding factors are a common feature of complex transcriptional networks, enabling context-dependent modulation of gene expression. Furthermore, to examine the functional relationship between SlNAC2 and SlDREB2, we generated transgenic lines overexpressing *SlNAC2* while simultaneously silencing *SlDREB2* (*OE-SlNAC2/TRV-SlDREB2*). Comparative analysis between *OE-SlNAC2/TRV-SlDREB2* and *TRV-Control* lines under saline–alkali stress revealed that knock down of *SlDREB2* function exacerbates stress susceptibility in the context of *SlNAC2* overexpression, as indicated by severe leaf wilting. In addition, phenotypic analyses were performed on recently obtained homozygous *SlDREB2* overexpression (OE) line. The OE line exhibited enhanced salt–alkali tolerance compared to WT, providing additional confirmation of the genetic relationship with SlNAC2. Collectively, the data support a model in which SlNAC2, through its interaction with SlDREB2, forms a heterodimeric regulatory complex that orchestrates the expression of melatonin-related genes and ROS-associated gene, ultimately modulating tomato tolerance to saline–alkali environments.

As a conceptual summary, we developed a hypothetical framework to illustrate how the SlNAC2–SlDREB2-melatonin regulatory module cooperatively governs saline–alkali stress resilience in tomato. Following saline–alkali treatment, SlNAC2 exhibits dual transcriptional activities: it functions as an activator of *SlCV*, resulting in ROS overaccumulation, while simultaneously serving as a repressor of *SlCOMT2* and *SlSNAT*, leading to diminished melatonin levels. This coordinated regulation ultimately heightens tomato susceptibility to stress. The interaction of SlNAC2 with SlDREB2 further refines these transcriptional outputs, underscoring the central role of the SlDREB2–SlNAC2 regulatory axis in orchestrating stress responses. The SlNAC2–SlDREB2 module functions as a central regulatory hub, integrating external stress signals with intrinsic developmental programs to maintain physiological homeostasis under adverse environmental conditions (Fig. 9). Given the persistent challenges in breeding cultivars with durable abiotic stress tolerance, SlNAC2 and SlDREB2 have emerged as promising molecular targets for engineering tomato resilience to saline–alkali stress and potentially other environmental stresses, providing a foundation for future crop improvement strategies.

## Materials and methods

### Plant materials and growth conditions

All transgenic and wild-type lines employed in this study were generated in the genetic background of *Solanum lycopersicum* cv. Micro-Tom. For transient transformation analyses, *N. benthamiana* was selected as the model plant. To construct *SlNAC2* overexpression plants, the full-length coding sequence of *SlNAC2* was isolated from Micro-Tom cDNA and ligated into a linearized *pCAMBIA2301-S2* backbone vector using a commercially available cloning kit (Vazyme, China), following the manufacturer’s guidelines, to generate the *SlNAC2* overexpression construct suitable for plant transformation. For RNAi-mediated gene knockdown, 300-bp gene-specific fragment of *SlNAC2* was incorporated flanking a PDK gene intron within the pCAMBIA2301 vector to form an inverted-repeat RNAi cassette, under the regulatory element of the CaMV 35S promoter and OCS terminator. Following their initial introduction into *Agrobacterium tumefaciens* strain EHA105, the plasmid constructs described above were subsequently delivered into tomato cotyledons using an *Agrobacterium*-mediated transformation method [17]. In VIGS-based gene silencing assays, some targeted fragments of *SlNAC2*, *SlCOMT2*, and *SlDREB2* were cloned into the pTRV2 vector to form the recombinant plasmids, which were then used to specifically suppress the expression of target genes in plant tissues [86, 87]. To achieve transient overexpression plants, the coding sequence of *SlNAC12* was cloned into a PVX-based vector and introduced into tomato plants by agroinfiltration. Plants infected with empty PVX vector were used as controls [88, 89].

After surface sterilization in 10% sodium hypochlorite solution, seeds from different genotypes were cultured on Murashige and Skoog (MS) medium supplemented with 4.4 g/l MS, 0.5 g/l MES, 20 g/l sucrose, and 1% agar (pH 5.8). The plates were then incubated for 10 days in a growth chamber at 28°C with a 16-h light/8 h dark photoperiod and 200 μmol m^−2^ s^−1^ photons. To ensure uniform development prior to analysis, seedlings from the respective lines were transferred into soil and subjected to consistent environmental conditions for subsequent assessment. Additionally, leaves obtained from *N. benthamiana* plants at the 5-week developmental stage were utilized in transient expression experiments.

### Evolutionary relationship analysis of SlNAC2

A total of 13 protein sequences, comprising SlNAC2 and 12 homologous proteins, were analyzed phylogenetically based on their amino acid sequences to investigate their evolutionary divergence. Following alignment of the selected sequences via the maximum likelihood (ML) method, a rootless phylogenetic tree was constructed using MEGA (Molecular Evolutionary Genetic Analysis) 7 software. To improve the accuracy of the phylogenetic tree, bootstrap analysis with 1000 replicates was performed, and any alignment gaps and ambiguous residues were completely removed.

### RNA extraction and qRT-PCR analysis

According to the manufacturer’s instructions, the extraction of total RNA from tomato cultivar Micro-Tom was conducted with the M5 Total RNA Extraction Reagent Kit (Mei5bio, Beijing, China). cDNA synthesis was carried out using the HiScript III RT SuperMix kit (Vazyme, Nanjing, China), which contains a gDNA wiper to improve the efficiency and accuracy of the reaction. In brief, a total of 1-μg RNA was incubated with 4× gDNA wiper reagent for genomic DNA removal, followed by reverse transcription using 5× HiScript III qRT SuperMix provided in the kit. qRT-PCR was performed through SYBR Green Master Mix on Thermal Cycler Dice® Real-Time System III (TaKaRa, Kusatsu, Japan), strictly following the method described in previous studies [17, 70, 90]. *SlActin2* served as the internal reference gene, and relative gene expression levels were determined using the 2^−ΔΔCT^ method. The primers used for qRT-PCR are listed in Table S1.

### Subcellular localization of SlNAC2 protein

To investigate the localization of SlNAC2 *in vivo*, a GFP fusion vector was constructed by inserting the *SlNAC2* CDS into the *2301-S2-GFP* plasmid backbone. The construct was then introduced into *A. tumefaciens* GV3101 for further analysis. The recombinant plasmid *2301-S2-SlNAC2-GFP* and a nucleus-localized marker were transiently co-expressed in 5-week-old *N. benthamiana* leaves through *Agrobacterium*-mediated co-infiltration for subsequent observation. Following dark treatment for 24 h and cultivation under long-day conditions (16 h light/8 h dark) for 2 additional days, plants were imaged for fluorescence using a laser confocal microscope (LEICA TCS SP5II, Wetzlar, Germany).

### Phenotypic evaluation of saline–alkali stress tolerance

Homozygous T3 transgenic lines overexpressing SlNAC2, as well as RNAi-mediated knockdown for these genes, together with their wild-type (WT) counterparts, were used for evaluating resistance under salt–alkali conditions. Seeds from the indicated genotypes were germinated on filter paper moistened with 100 mM Na_2_CO₃ solution, and germination rates were recorded after a 5-day incubation to evaluate the impact of saline–alkali stress on seed germination. To assess root growth under saline–alkali conditions, different genotype seeds were initially germinated for 2 days in standard Hoagland solution (pH 6.8), followed by a 6-day treatment in Hoagland solution containing 0 or 100 mM Na_2_CO_3_ (pH 8.7), after which root lengths were measured. For phenotypic characterization under salt–alkali stress, 4-week-old plants of various genotypes were irrigated every 3 days with either a saline- alkali solution (500 mM Na_2_CO_3_ in Hoagland medium) or standard Hoagland solution (control). Plants subjected to salt–alkali stress were additionally treated with 0 or 100 μM melatonin (MT) via foliar spray to determine its effect on stress tolerance [60]. This treatment was maintained over a 16-day period. Thereafter, comprehensive phenotypic observations were carried out for all genotypes, and the corresponding physiological and biochemical parameters were documented for comparative analysis.

### Physiological and biochemical measurements

Malondialdehyde (MDA) content was determined using a TBA assay, in which leaf sections were incubated in a TBA/TCA reaction mixture (0.6%/10%). Following a 15-min heat treatment at 100°C, the samples were cooled, after which absorbance was measured at the specified wavelengths of 532, 600, and 450 nm to calculate MDA concentrations. For ion leakage analysis, leaf tissues were submerged in deionized water, followed by vacuum infiltration for 30 min, and the initial conductivity (E1) of the resulting solution was measured. Subsequently, the leaf samples were exposed to a 10-min boiling treatment, allowed to cool to room temperature, and the second conductivity (E2) of the solution was recorded. As a final calculation, the ion leakage rate was derived by applying the formula (E1/E2) × 100. Hydrogen peroxide (H_2_O_2_) and superoxide anion radical (O_2_^−^) levels were determined through the experimental approaches previously described by Alexieva *et al.* and Hou *et al.*, respectively [91, 92]. To assess the accumulation of ROS in tomato leaf tissues, including H_2_O_2_ and O_2_^−^, 3,3′-diaminobenzidine (DAB) and NBT histochemical staining were performed, allowing both visualization and quantification. Leaves obtained from selected plant lines were treated with DAB or NBT for 40 min and 2 h, respectively, decolorized in 95% ethanol, and then transferred to 60% glycerol for subsequent image capture. To prepare samples for enzymatic activity analysis, a total of 1 g of freshly harvested leaf material was homogenized in 3 ml of extraction buffer solution consisting of 50 mM sodium phosphate (pH 7.8), 2% (w/v) PVP, and 1 mM EDTA. The homogenates underwent centrifugation (12 000 × *g*, 20 min, 4°C), and the supernatants collected thereafter served as templates for superoxide dismutase (SOD), peroxidase (POD), and catalase (CAT) activity measurements. Enzyme activities of SOD, POD, and CAT were determined using commercial assay kits (Nanjing Jiancheng), following the manufacturers’ instructions. Measurement of melatonin was performed based on the standardized method previously established by Park *et al.* [93], with slight modifications. Briefly, ~0.3 g of fresh leaf samples were homogenized in 4 ml of sodium phosphate buffer (50 mM, pH 7.4), and the homogenates were centrifuged at 3000 rpm for 20 min to isolate the supernatant for melatonin determination. Melatonin levels were assessed using the Plant Melatonin (MT) ELISA Kit (Shanghai Enzyme-linked Biotechnology Co., Ltd, Shanghai, China), and absorbance reading at 450 nm were obtained with a multimode plate reader (Agilent BioTek CYTATION1, USA).

### GUS staining

Detached plant tissues were incubated in GUS staining solution at 37°C for 24 h, followed by decolorization in 75% ethanol for an additional 24 h, and subsequently stored in 50% glycerol for microscopic observation. The histochemical GUS staining buffer was prepared by mixing 2 ml of 50 mmol/l K_3_[Fe(CN)_6_], 2 ml of 50 mmol/l K_4_[Fe(CN)_6_], 4 ml of 0.5 mol/l EDTA (pH 8.0), 5 ml of methanol, 100 mg of X-Gluc, and 0.2 ml of Triton X-100, and the final volume was brought to 200 ml with 50 mM phosphate buffer.

### Y1H assays

As previously described [17], the CDS fragment of *SlNAC2* was ligated into the vector *pGADT7*, while the corresponding promoter elements were incorporated into the reporter vector pHIS2.1. The resulting plasmids, together with a control empty vector, were introduced into yeast cells Y1HGold and subsequently grown on SD/-Leu-Trp and SD/-His-Leu-Trp medium containing 50 mM 3-AT. The DNA-binding activity of SlNAC2 was assessed by monitoring yeast cell growth over a 3-day period. The primers utilized for Y1H are listed in Table S1.

### Y2H assays

In Y2H experiments, the CDS segment of *SlNAC2* was cloned into the *pGBKT7* vector, while that of *SlDREB2* was inserted into the pGADT7 vector, generating the fusion constructs *pGBKT7-SlNAC2* and *pGADT7-SlDREB2*. Subsequently, these recombinant plasmids were co-transformed into Y2HGold yeast cells and cultured on SD/-Leu-Trp-Ade-His selective medium. The primers used for Y2H are listed in Table S1.

### BiFC assay

To investigate whether SlNAC2 interacts with SlDREB2 and SlDREB3, a bimolecular fluorescence complementation (BiFC) assay was carried out following established protocols [17]. In brief, recombinant constructs containing SlNAC2-cYFP, SlDREB2-nYFP, and SlDREB3-nYFP were generated and transferred into *Agrobacterium* strain GV3101, then simultaneously infiltrated into *N. benthamiana* leaves through the *Agrobacterium*-based transformation method. YFP signal observation was ultimately executed using a LEICA TCS SP5II laser confocal microscope (Wetzlar, Germany).

### Dual-luciferase assay

To construct the reporter plasmids, PCR-based amplification of target gene promoters was performed using tomato genomic DNA, and the resulting fragments were inserted into pGreenII0800-LUC backbone. In parallel, the effector construct was assembled by ligating the CDS of *SlNAC2* into pGreenII 62-SK vector, and this fusion construct, along with the reporter plasmids, was then delivered into *N. benthamiana* leaves (5-week-old) via *Agrobacterium*-mediated infiltration. Luciferase activity (LUC/REN ratio) was quantified using the Dual-Luciferase Reporter Assay System Kit (Promega, Madison, WI, USA), according to the instructions provided by the manufacturer.

### ChIP assay

To investigate the potential interaction between SlNAC2 and tomato genomic DNA, chromatin immunoprecipitation (ChIP) followed by quantitative PCR was conducted using the EpiQuik Plant ChIP Kit (Epigentek, NY, USA) according to the provided protocol. Generally speaking, 3-week-old seedlings from the designated lines were incubated in Hoagland solution containing 200 mM Na_2_CO₃ for 24 h, followed by crosslinking of protein and DNA with 1% formaldehyde. The protein–DNA complexes, obtained from cell lysis and chromatin fragmentation, were immunoprecipitated using an anti-GFP antibody (Abmart, Shanghai, China), with immunoglobulin G (IgG) serving as the negative control. Ultimately, quantitative PCR was performed to examine SlNAC2’s DNA-binding efficiency, and the relative DNA enrichment was determined according to previously described methods [23]. The primers used for the ChIP-qPCR analysis are listed in Table S1.

### Electrophoretic mobility shift assays

For EMSA, the full-length coding region of *SlNAC2* was cloned into the vector pET-28a to generate a His-tagged SlNAC2 recombinant protein, which was subsequently purified with the Ni Sepharose High Performance resin (GE Healthcare) following the manufacturer’s instructions. Protein–DNA interaction analysis was then executed through a chemiluminescent EMSA Kit from Beyotime (China). All biotin-tagged DNA probes used in the assay were commercially synthesized by Sangon Biotech (Shanghai, China), and the primer sequences used are provided in Table S1.

### Western blot analysis

To extract total proteins, 10-day-old seedlings from the specified genotypes were homogenized in RIPA buffer (Coolaber, China) containing 1 mM PMSF. After electrophoretic separation through 10% SDS-PAGE, the resulting proteins were then transferred onto PVDF membranes (Millipore, USA) for further analysis. To block nonspecific protein–antibody interactions, the PVDF membranes were treated with 5% milk in 1× TBST buffer supplemented with 0.02% Tween-20, and incubated at room temperature for 2 h. Afterward, these membranes were exposed to primary antibodies at 4°C overnight, followed by extensive washing in 1× TBST solution to remove unbound antibodies. Subsequently, secondary antibody incubation was performed at ambient temperature for 2 h using HRP-conjugated antibodies, and the protein signals were visualized with BeyoECL Plus detection system (Mei5bio, Beijing, China).

### Statistical analysis

To verify statistical comparison among different treatments, a Student’s *t* test was conducted. Each experiment was replicated three times, with significance levels denoted by asterisks (^*^P < 0.05; ^**^P < 0.01; ^***^P < 0.001).

## Supplementary Material

Web_Material_uhag029

## Data Availability

The data underlying this article are available in the article and in its online supplementary material.
