# Supplementary material for: Tomato NAC2-DREB2 module fine-tunes saline–alkali stress sensitivity via modulation of melatonin biosynthesis and ROS homeostasis
Source: Hortic Res. 2026 Jan 30;13(5):uhag029. doi: 10.1093/hr/uhag029 (PMC13148167; doi:10.1093/hr/uhag029)
Supplement: Web_Material_uhag029 [file web_material_uhag029.zip › SlNAC2-è¡¥å..å>_-V1.pdf]

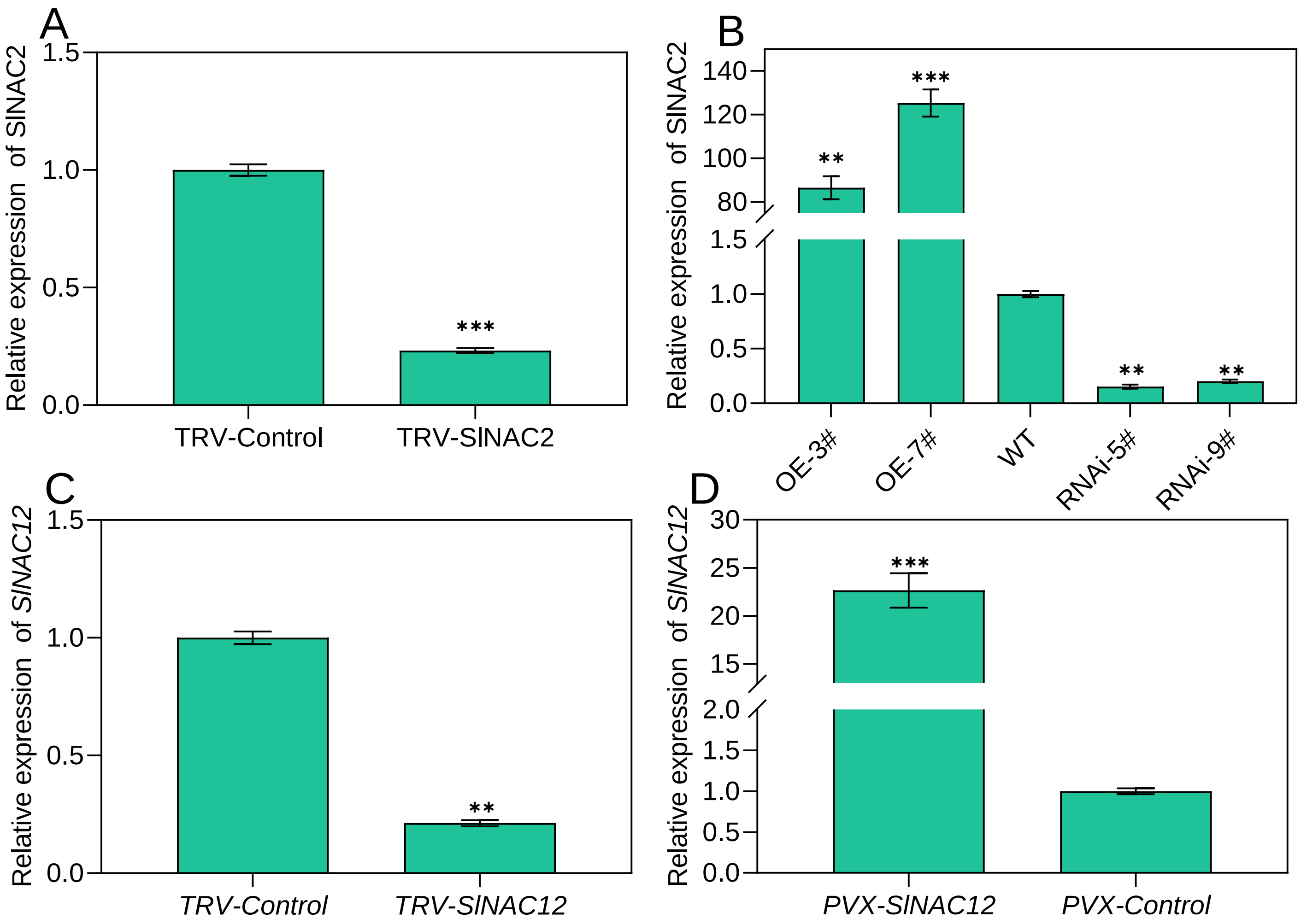

Figure S1. Characterization of *SlNAC2* and SlNAC12 transgenic lines. (A-B)The relative expression level of *SlNAC2* in transgenic lines. (C-D) Expression level of *SlNAC12* in PVX-based PVX-based transient overexpression plants, and TRV-mediated VIGS lines. *SlActin2* was utilized as the internal reference gene. The values represent the mean ± SD obtained from three independent biological replicates. Asterisks indicate significant differences levels (Student's t-test, ns, not significant, ***P* < 0.01, ****P* < 0.001) with corresponding controls.

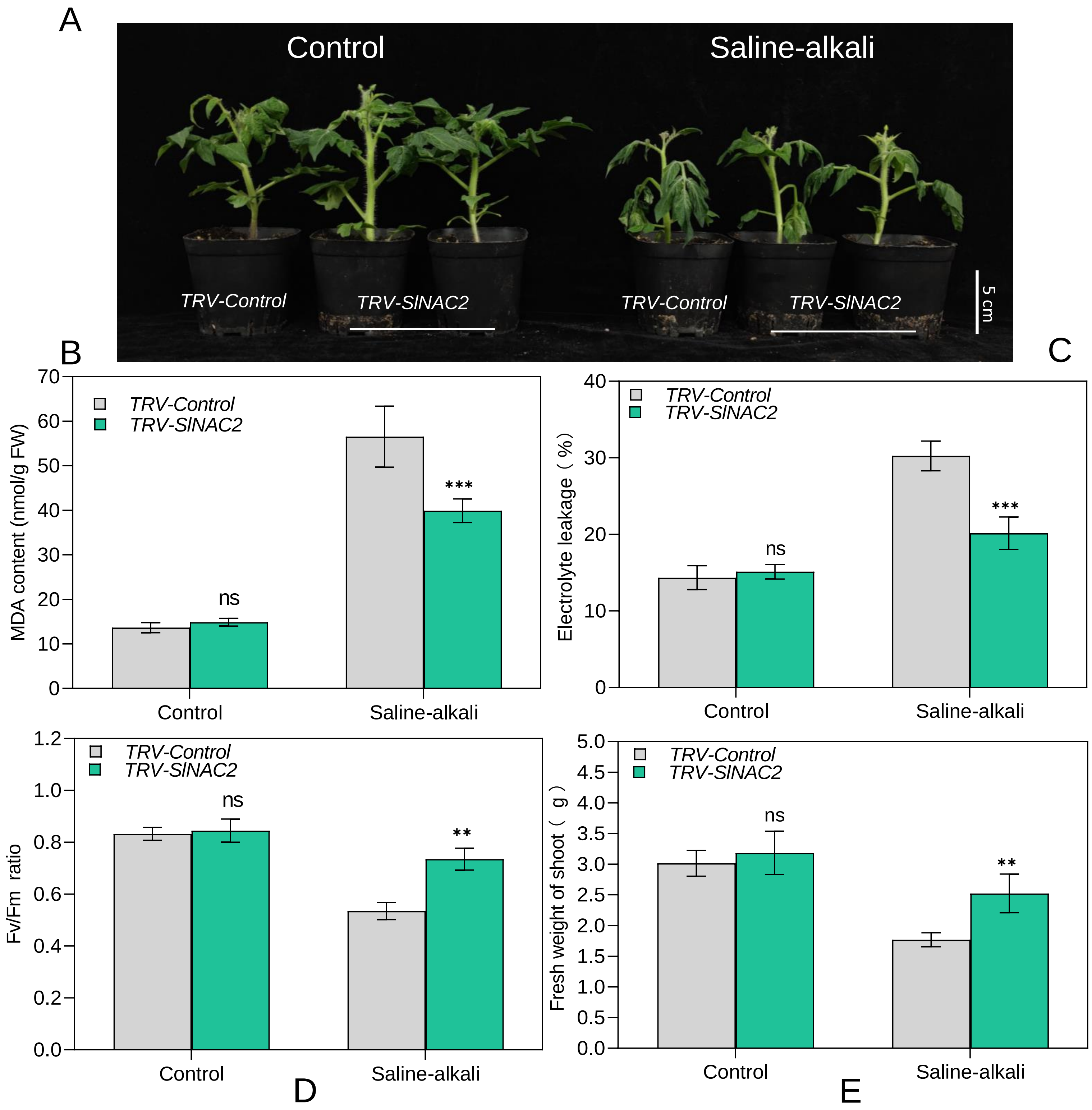

Figure S2. VIGS-mediated silencing of *SlNAC2* improves saline-alkali stress resistance in tomato. (A) Phenotypic observation of *SlNAC2*-silenced plants and control plants (bar=5 cm). 4-week-old seedlings were subjected to either normal growth conditions (Control) or 500 mM Na_2CO_3 stress for 5 days. (B-F) MDA content (B), Fv/Fm (C), ion leakage (D), and fresh weight (E). The values represent the mean ± SD obtained from three independent biological replicates. Asterisks indicate significant differences levels (Student's t-test, ns, not significant, ***P* < 0.01, and ****P*< 0.001) with corresponding controls.

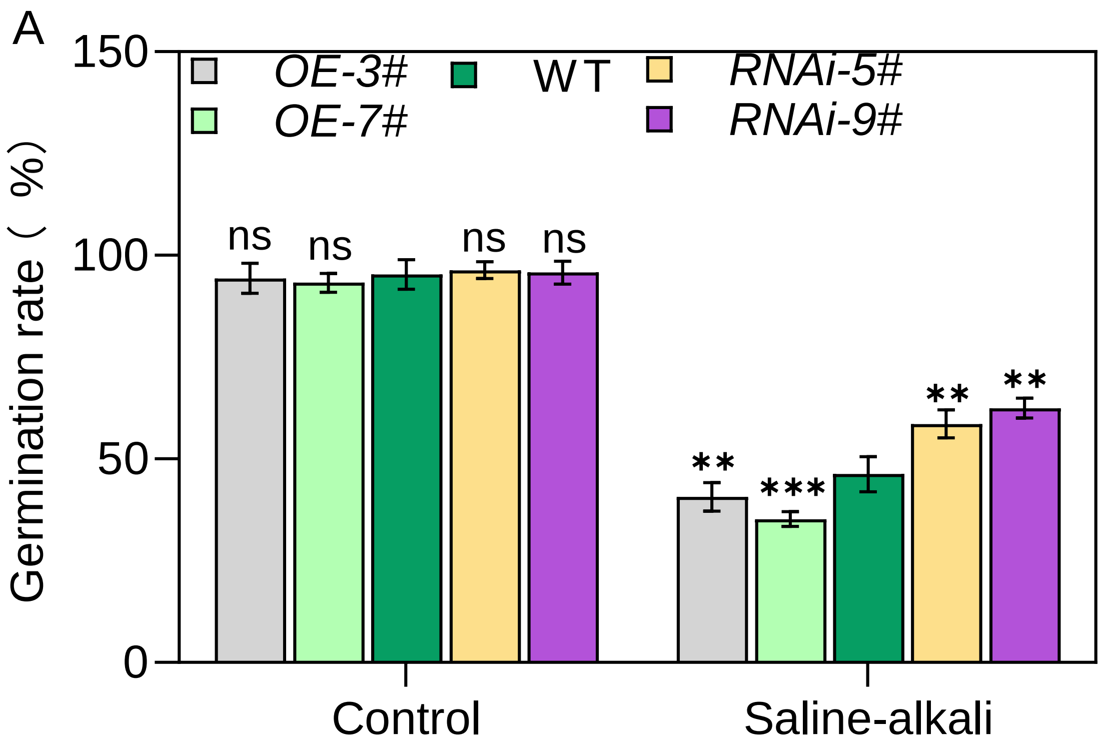

Figure S3. Statistical evaluation of seeds germination rate of WT, *OE-SlNAC2* and *RNAi-SlNAC2* lines. Seeds of these plants placed on Petri dishes with filter paper moistened without (Control) or with 100 mm Na_2CO_3 for 5 d. Data represent the means (± SD) of three independent biological replicates. Asterisks indicate statistically significant differences (ns, not significant, **P*<0.05, ***P*<0.01 by the student's test) from the corresponding control.

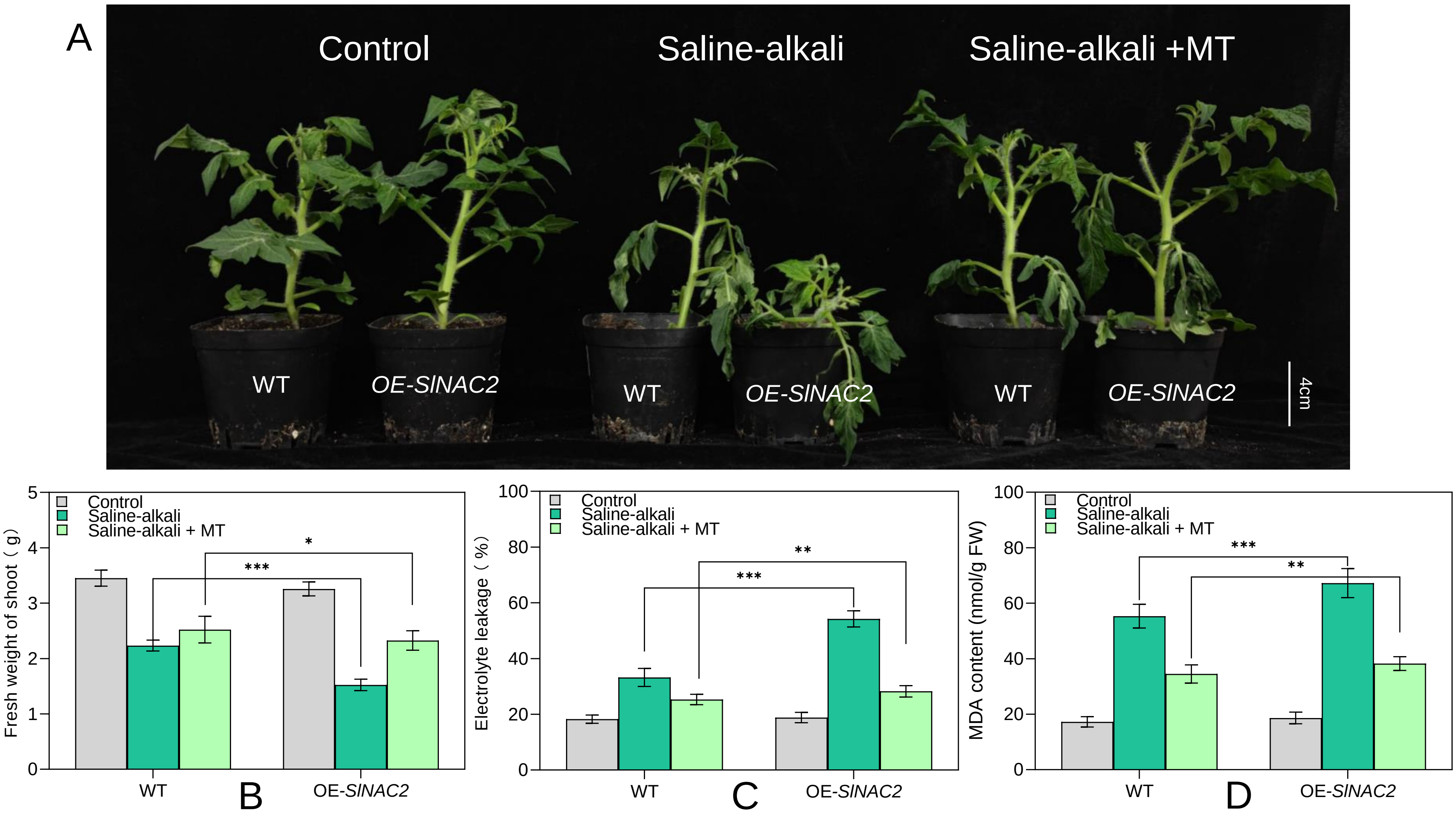

Figure S4. Exogenous MT partially alleviates the salt–alkali sensitivity of SlNAC2-overexpressing plants. (A) Phenotypic comparison of WT and *OE-SlNAC2* plants under control conditions, salt–alkali stress, and salt–alkali stress supplemented with MT. (B-D) Measurements of stress-related physiological parameters in WT and OE-SlNAC2 plants (A), fresh weight (B), ion leakage (C), MDA content (D). Data represent the means (± SD) of four independent biological replicates. Asterisks indicate statistically significant differences (ns, not significant, **P*<0.05, ***P*<0.01 by the student's test) from the corresponding control.

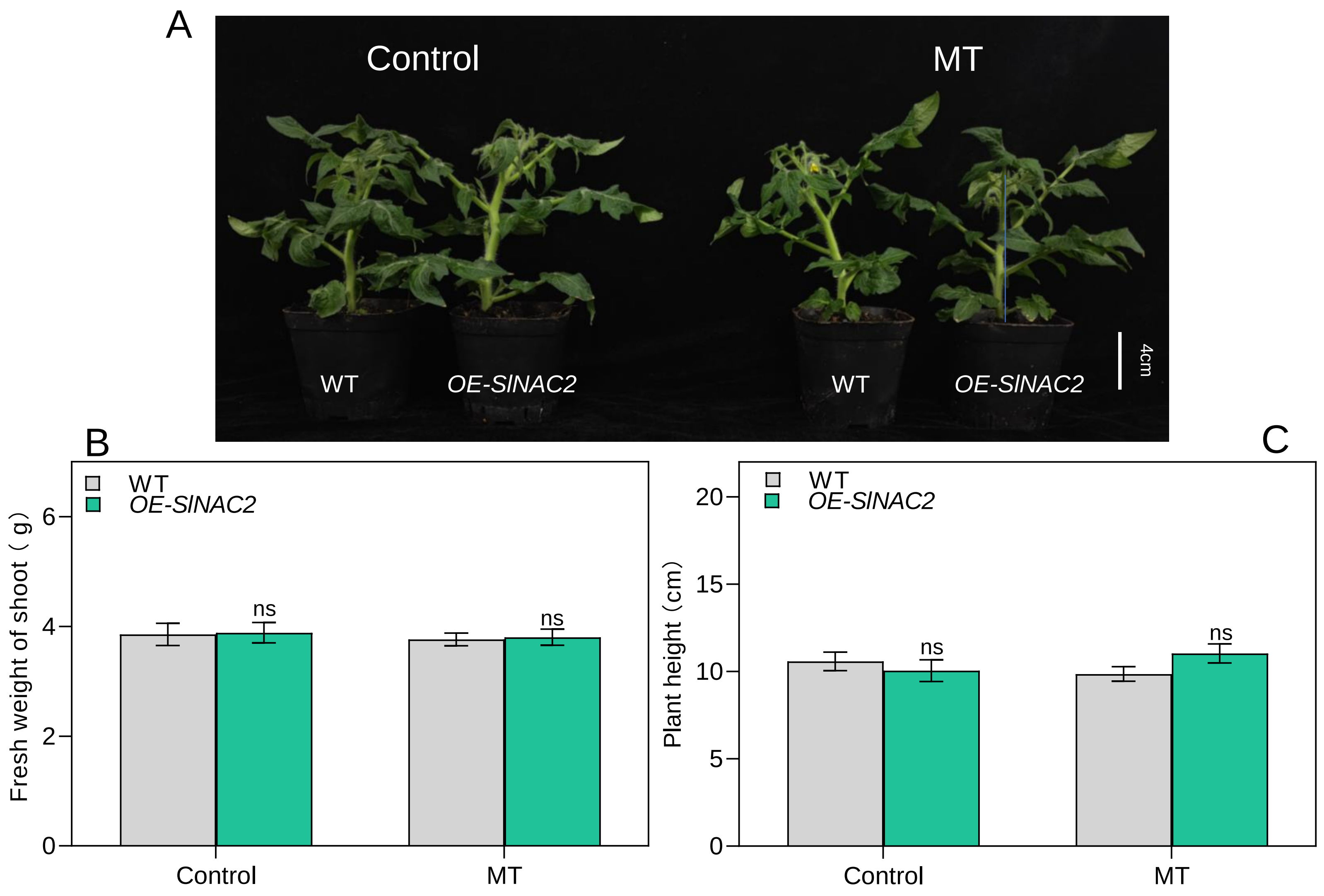

Figure S5. Effect of exogenous melatonin (MT) on growth and development of OE-SlNAC2 plants under non-stress conditions. (A) Phenotypic comparison of WT and *OE-SlNAC2* plants under control conditions with MT（100 μM） treatment. (B-C) Measurements of physiological parameters in WT and OE-SlNAC2 plants (A), fresh weight (B), plant height (C). Data represent the means (± SD) of four independent biological replicates. Asterisks indicate statistically significant differences (ns, not significant, by the student's test) from the corresponding control.

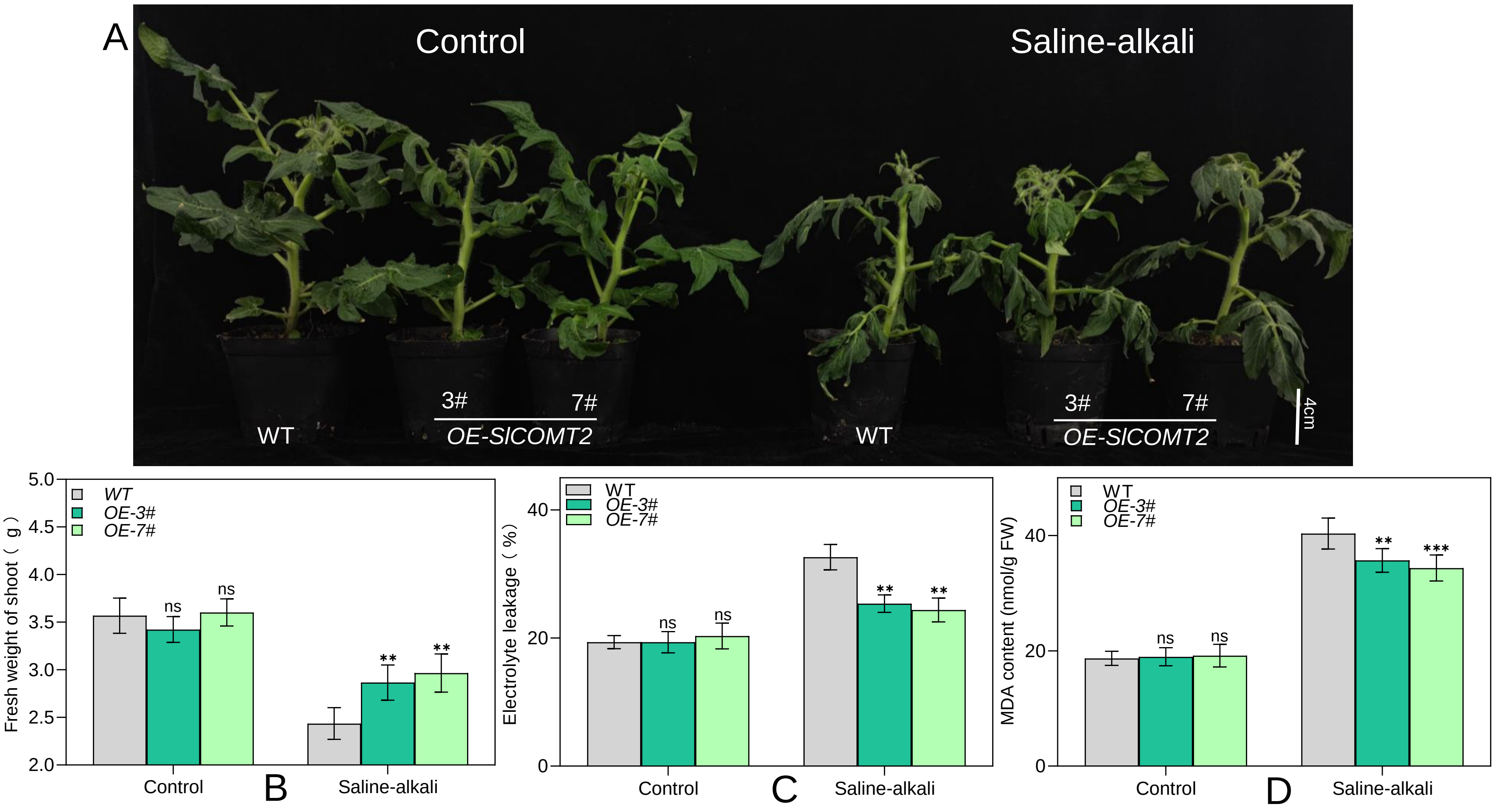

Figure S6. Phenotypic characterization of *SlCOMT2*-overexpressing plants under salt–alkali stress. (A) Phenotypic comparison of WT and *OE-SlCOMT2* plants under Saline-alkali treatment. (B-C) Measurements of physiological parameters in WT and *OE-SlCOMT2* plants (A), fresh weight (B), electrolyte leakage (C), MDA content (D). Data represent the means (± SD) of four independent biological replicates. Asterisks indicate statistically significant differences (ns, not significant, **P*<0.05, ***P*<0.01, ****P*<0.001 by the student's testby the student's test) from the corresponding control.

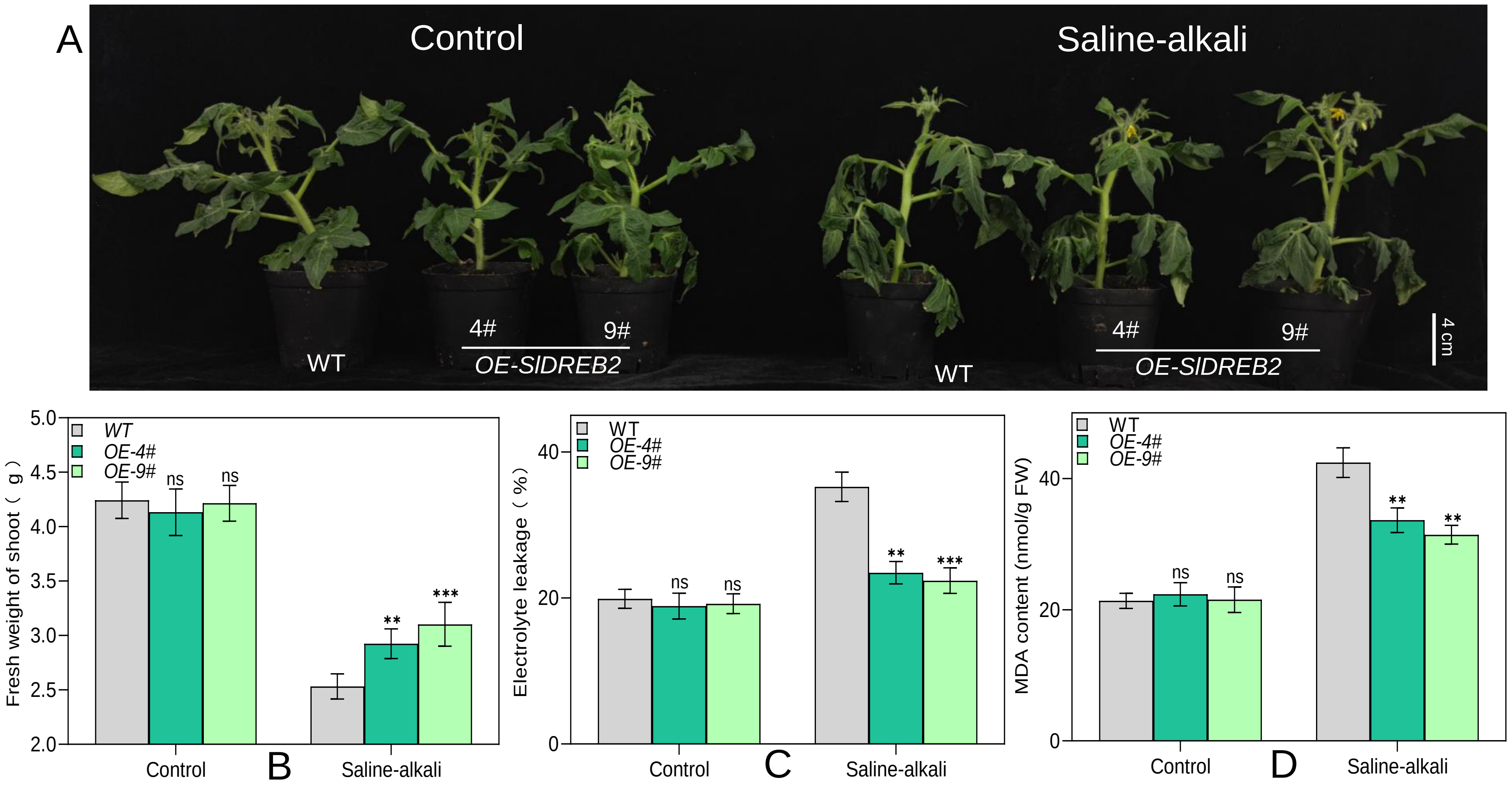

Figure S7. Phenotypic characterization of *SlDREB2*-overexpressing plants under salt–alkali stress. (A) Phenotypic comparison of WT and *OE-SlDREB2* plants under Saline-alkali treatment. (B-C) Measurements of physiological parameters in WT and *OE-SlDREB2* plants (A), fresh weight (B), electrolyte leakage (C), MDA content (D). Data represent the means (± SD) of four independent biological replicates. Asterisks indicate statistically significant differences (ns, not significant, ***P*<0.01, ****P*<0.001 by the student's testby the student's test) from the corresponding control.

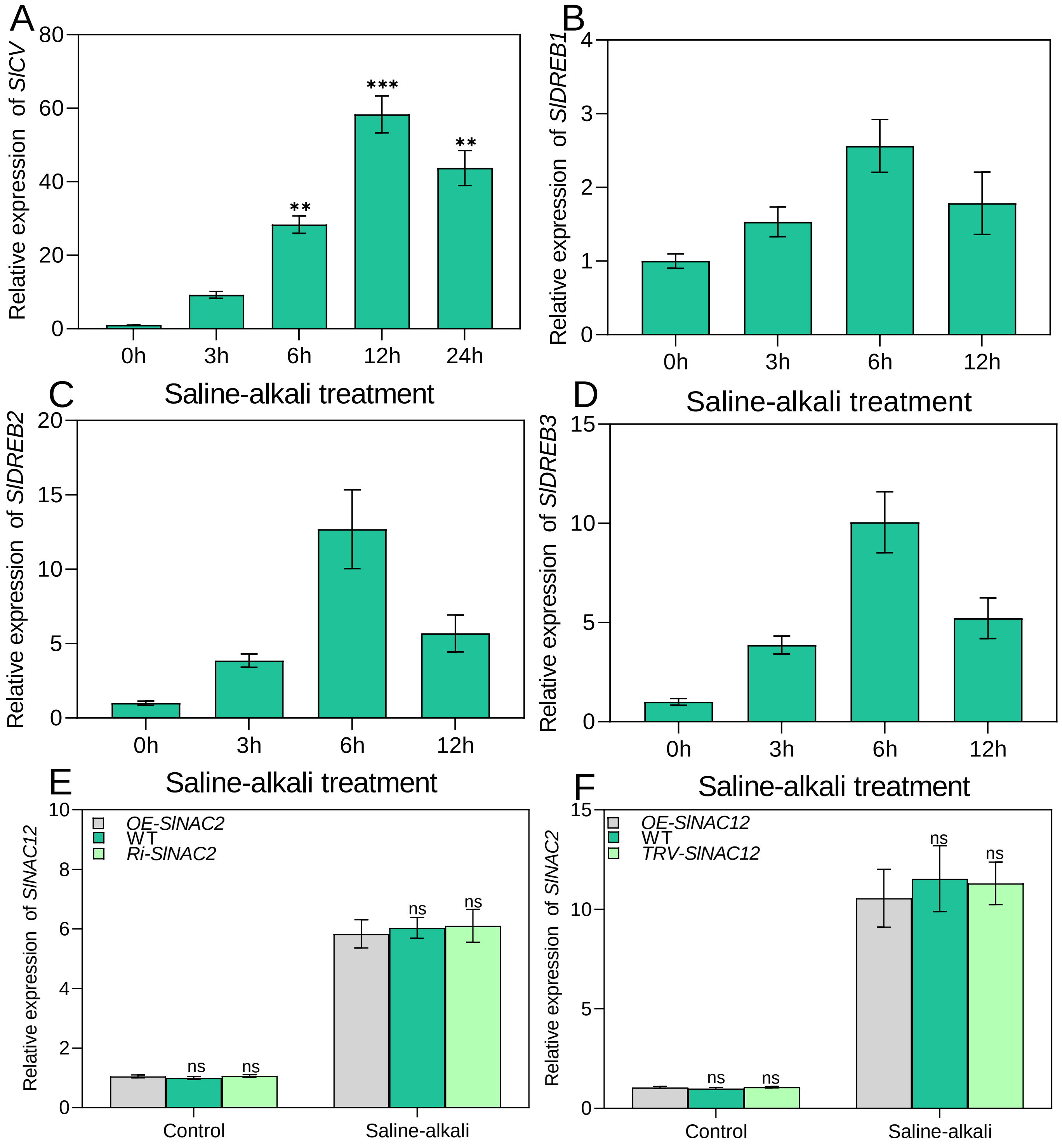

Figure S8. Expression pattern of *SlCV*, *SlDREB1*, *SlDREB2*, and *SlDREB3* under saline-alkali stress, and reciprocal expression analysis of SlNAC2 and SlNAC12 in transgenic lines.

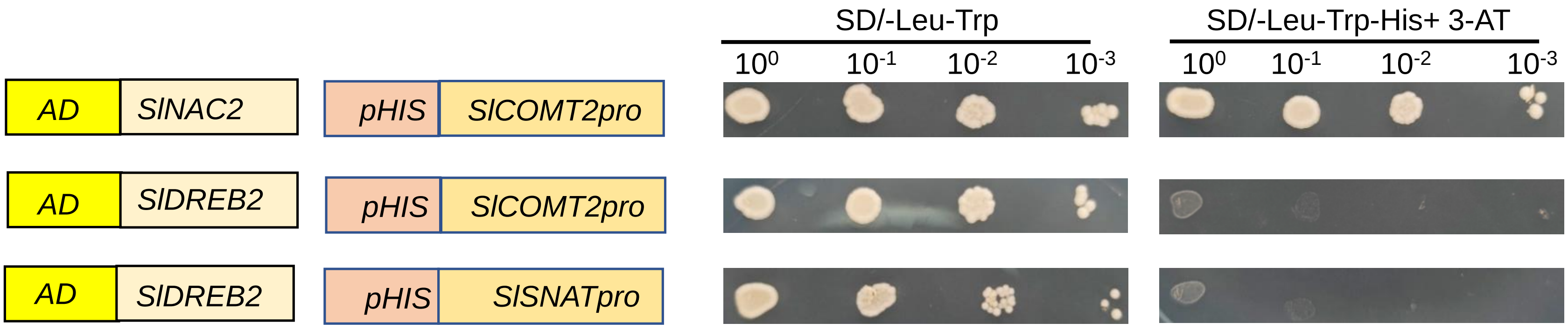

Figure S9. Y1H assays showed that SlDREB2 does not directly bind the promoters of *SlCOMT2* and *SlSNAT*.

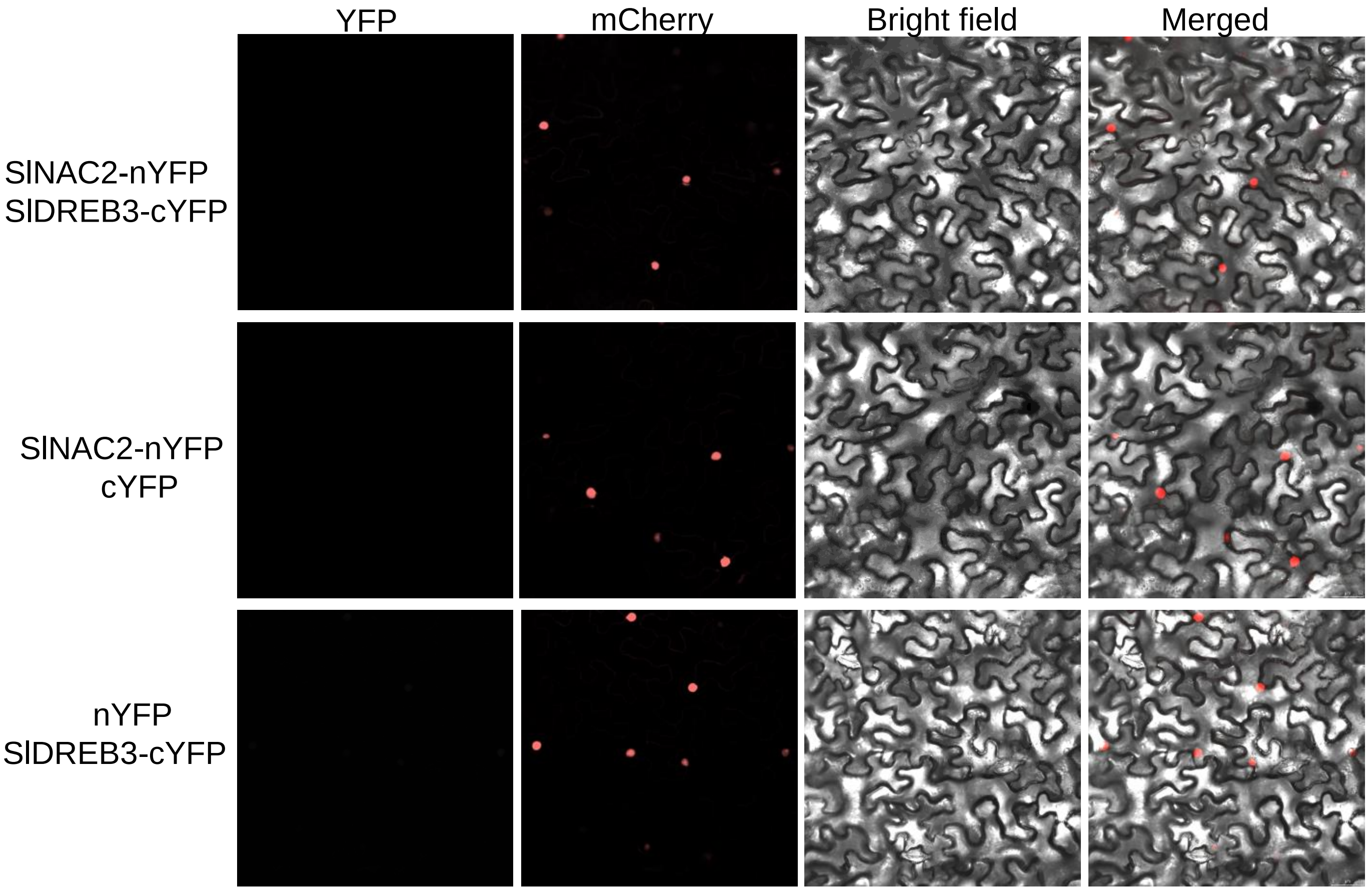

Figure S10. BiFC assays showed that SlDREB3 does not physically interact with SlNAC2 in cells, BES1n-mCherry, a nuclear marker.

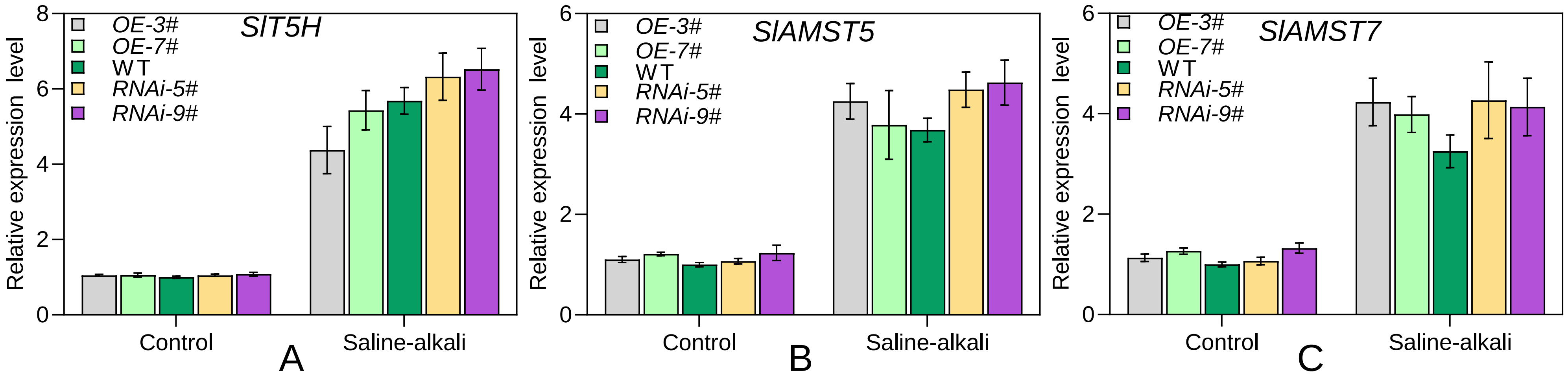

Figure S11. Transcript profiles of *SlT5H*, *SlAMST5*, and *SlAMST7* in WT, OE-SlNAC2, and RNAi-SlNAC2 plants during salt-alkali stress.
