# Supplementary material for: Tomato NAC2-DREB2 module fine-tunes saline–alkali stress sensitivity via modulation of melatonin biosynthesis and ROS homeostasis
Source: Hortic Res. 2026 Jan 30;13(5):uhag029. doi: 10.1093/hr/uhag029 (PMC13148167; doi:10.1093/hr/uhag029)
Supplement: Web_Material_uhag029 [file web_material_uhag029.zip › Table-å1⁄4ç%c-V1.pdf]

| Table S1 Primers used in this study. | |
|---|---|
| Primer | sequence（5'-3'） |
| **For generating Transgenic plants** | |
| SlNAC2-OE F | tctctcttctcaagcttggatcc**atggaaattgtgtgtggatttggt** |
| SlNAC2-OE R | attaaagcagggcatgcctgcag**tcaataattccatagacaatcgat** |
| SlDREB2A-OE F | tctctcttctcaagcttggatcc**atggctgtgcttgatcgaacttc** |
| SlDREB2A-OE R | attaaagcagggcatgcctgcag**ttagaaatccaagacatccaactc** |
| RNAi-SlNAC2 F | gactgatgaagagcttataacac |
| RNAi-SlNAC2 R | gaatatttccttatctttgcctg |
| TRV-SlDREB2A F | tgtgagtaaggttaccgaattcgatgaggctaagcaggtgttagat |
| TRV-SlDREB2A R | gcgtgagctcggtaccggatccgatatgaaggatcataggcatac |
| TRV-SlNAC2 F | tgtgagtaaggttaccgaattcgactgatgaagagcttataacac |
| TRV-SlNAC2 R | gcgtgagctcggtaccggatccgaatatttccttatctttgcctg |
| TRV-SlCOMT2 F | tgtgagtaaggttaccgaattcTGTCTGGAGTTTCTGTGCCA |
| TRV-SlCOMT2 R | gcgtgagctcggtaccggatccCAACCACCTCAGGCAAATCA |
| **For qRT-PCR** | |
| SlActin2 F | TTGCTGACCGTATGAGCAAG |
| SlActin2 R | GGACAATGGATGGACCAGAC |
| SlCV-F | ATCGCCGGAGACATGCAATTACA |
| SlCV-R | TTCCCATCTCCGGCGAGTCACCG |
| SlNAC2 F | gactgatgaagagcttataacac |
| SlNAC2 R | gaatatttccttatctttgcctg |
| SlCOMT1 F | CTTCTTGCTGCTTACTCTGTTC |
| SlCOMT1 R | CCATCGGCATTCTTAGTCA |
| SlCOMT2 F | TGTCTGGAGTTTCTGTGCCA |
| SlCOMT2 R | CAACCACCTCAGGCAAATCA |
| SlCBL10 F | CCATTATTGCACTCATTGAAGC |
| SlCBL10 R | AGCAAGGTCCGTGTATTCGT |
| SlSOS1 F | TCGAGTGATGATTCTGGTGG |
| SlSOS1 R | GACGCCTTTCCACACTCTGAT |
| SlSOS2 F | CTGCTTAGGACAAGGACTCG |
| SlSOS2 R | CTGCTTAGGACAAGGACTCG |
| SlDREB2A F | GAGAAATTAGACGCGTGTGATG |
| SlDREB2A R | CAGCAACCCATTTACCCCATG |
| SlHKT1;1 F | TCTAGCCCAAGAAACTCAAAT |
| SlHKT1;1 R | CTAATGTTACAACTCCAAGGAATT |
| SlSNAT F | TCAGGGACAAGGACTTGGAA |
| SlSNAT R | TTCCCTCTGGATCAGGTTCA |
| SlT5H F | TCGGCCAAATTCCGACTCTA |
| SlT5H R | GAGCAACCGAAGGAGAGGTA |
| SlAMST5 F | CGAAGCTATGGGATGTGACG |
| SlAMST5 R | CCTTCCAAGCCTTCAACCAC |
| SlAMST7 F | GACGTCATTGGTGGACATCG |
| SlAMST7 R | TGCGTTGGCATGAGGAATTT |
| SlNAC12 F | ATGATGGAAGTGGAAAAAAT |
| SlNAC12 R | TCAAACAAAGGATCAAACGAAA |
| **For subcellular localization** | |
| SlNAC2-GFP F | tcgactctagaaagcttctgcag**atggaaattgtgtgtggatttggt** |
| SlNAC2-GFP R | accatggtaccggatccactag**tataattccatagacaatcgat** |
| **For Dual-Luc reporter assay** | |
| SlCOMT2p-0800 F | aggtcgacggtatcgataagcttAAAATCTCACAAAATTAATTGAGA |
| SlCOMT2p-0800 R | ctagaactagtggatcccccgggGAATTTAATATTAATACTTTGTA |
| SlSNATp-0800 F | aggtcgacggtatcgataagcttAAGGTCCATTCCTATCCCCACT |
| SlSNATp-0800 R | ctagaactagtggatcccccgggAAGAAAGAAGAATTTAAATAAAT |
| SlCVp-0800 F | aggtcgacggtatcgataagcttTTGAAATAAGAATCACAAGCTGA |
| SlCVp-0800 R | ctagaactagtggatcccccgggGTGAAAATATAAATTACATTACAT |
| SlNAC2-SK F | tctctcttctcaagcttggatcc**atggaaattgtgtgtggatttggt** |
| SlNAC2-SK R | attaaagcagggcatgcctgcag**tcaataattccatagacaatcgat** |

| | |
|---|---|
| **For ChIP-qPCR** | |
| P1-F | TTTTCAGCAATAGAAAGTATCT |
| P1-R | gataattaataaATTAAAACATT |
| P2-F | AAAATCTCACAAAATTAATTGAGA |
| P2-R | GAATTTAATATTAATACTTTGTA |
| P3-F | CAAAAAGAAGAGTTCAAAACTATT |
| P3-R | GATTTATTAGTCATTTATATTGC |
| P4-F | AAGGTCCATTCCTATCCCCACT |
| P4-R | AAGAAAGAAGAATTTAAATAAAT |
| P5-F | TTTGAATAATATTTTCTCTGAGT |
| P5-R | ATACTAGTAATAAGTAATTTTTT |
| P6-F | TTGAAATAAGAATCACAAGCTGA |
| P6-R | GTGAAAATATAAATTACATTACAT |
| P7-F | TATTGTTTCTAAATGATGAAGTG |
| P7-R | ATTTTGTACTTCTTTTATTGTTGAA |
| **For EMSA** | |
| SlCOMT2-Probe / Wild type competito | AAAATCTCACAAAATTAATTGAGA |
| SlCOMT2-Probe / Wild type competito | GAATTTAATATTAATACTTTGTA |
| SlCOMT2-Mut competitor F | AATTGGACATTTGAGTGACAAATATTTTTAAGGTTAAAATTAATAATGGGACGGAGGGATCC |
| SlCOMT2-Mut competitor R | GGATCCCTCCGTCCCATTATTAATTTTAACCTTAAAAATATTTGTCACTCAAATGTCCAATT |
| SlSNAT-Probe / Wild type competitor F | AAGGTCCATTCCTATCCCCACT |
| SlSNAT-Probe / Wild type competitor F | AAGAAAGAAGAATTTAAATAAAT |
| SlSNAT--Mut competitor F | AAAATTTTAAAAAATCTCAAAATATTTTTAGAGTATTGAACAAACAATTGAATAACAAAAACTTTATTTATTTATATTTCTTTTTCTTTTAAGTA |
| SlSNAT--Mut competitor R | TACTTAAAAGAAAAAGAAATATAAATAAATAAAGTTTTTGTTATTCAATTGTTTGTTCAATACTCTAAAAATATTTTGAGATTTTTTAAAATTTT |
| SlCV-Probe / Wild type competitor F | TTGAAATAAGAATCACAAGCTGA |
| SlCV-Probe / Wild type competitor R | GTGAAAATATAAATTACATTACAT |
| SlCV-Mut competitor F | AATTTTATCAATATTTTCTTAATTAACAAAAACTATTTTGGAGTTTTAAATAGTTTATTTAAGTCATTCCGT |
| SlCV-Mut competitor R | ACGGAATGACTTAAATAAACTATTTAAAACTCCAAAATAGTTTTTGTTAATTAAGAAAATATTGATAAAATT |
| SlNAC2-pET28a F | gacagcaaatgggtcgcggatcc**atggaaattgtgtgtggatttggt** |
| SlNAC2-pET28a R | ccgcaagcttgtcgacggagctc**tcaataattccatagacaatcgat** |
| **For Y1H and Y2H** | |
| SlCOMT2p-pHIS2.1 F | GAATTCAAAATCTCACAAAATTAATTGAGA |
| SlCOMT2p-pHIS2.1 R | GGATCCGAATTTAATATTAATACTTTGTA |
| SlSNATp-pHIS2.1 F | GAATTCAAGGTCCATTCCTATCCCCACT |
| SlSNATp-pHIS2.1 R | GGATCCAAGAAAGAAGAATTTAAATAAAT |
| SlCVp-pHIS2.1 F | GAATTCTTGAAATAAGAATCACAAGCTGA |
| SlCVp-pHIS2.1 R | GGATCCGTGAAAATATAAATTACATTACAT |
| SlNAC2-pGADT7 F | tggccatggaaattgtgtgtggatttggt |
| SlNAC2-pGADT7 R | tgcatcaataattccatagacaatcgat |
| SlNAC12-pGADT7 F | tggccatggaggccagtgaattcATGATGGAAGTGGAAAAAAT |
| SlNAC12-pGADT7 R | tgcagctcgagctcgatggatccTCAAACAAAGGATCAAACGAAA |
| SlDREB2A-pGBKT7 F | atatggccatggaggccgaattc**atggctgtgcttgatcgaacttc** |
| SlDREB2A-pGBKT7 F | tgctagttatgcggccgctgcag**ttagaaatccaagacatccaactc** |
| **For CoIP assays** | |
| SlDREB2A-MYC F | GTCGACatggctgtgcttgatcgaacttca |
| SlDREB2A-MYC R | GGTACCgaaatccaagacatccaactcatc |
| **For BiFC assays** | |
| SlNAC2-nYFP F | GGTACC**atggaaattgtgtgtggatttggt** |
| SlNAC2-nYFP R | GGATCC**ataattccatagacaatcgat** |
| SlDREB2A-cYFP F | GGATCC**atggctgtgcttgatcgaacttca** |
| SlDREB2A-cYFP R | CTGCAG**gaaatccaagacatccaactcatc** |
